# Clinical efficacy and safety of anti-PD-1/PD-L1 inhibitors for the treatment of advanced or metastatic cancer: a systematic review and meta-analysis

**DOI:** 10.1038/s41598-020-58674-4

**Published:** 2020-02-07

**Authors:** Leitao Sun, Leyin Zhang, Jieru Yu, Yinan Zhang, Xi Pang, Chenghao Ma, Minhe Shen, Shanming Ruan, Harpreet S. Wasan, Shengliang Qiu

**Affiliations:** 1 The First Clinical Medical College of Zhejiang Chinese Medical University, Hangzhou, 310053 Zhejiang China; 2 College of Basic Medical Science, Zhejiang Chinese Medical University, Hangzhou, 310053 Zhejiang China; 3 Department of Medical Oncology, The First Affiliated Hospital of Zhejiang Chinese Medical University, Hangzhou, 310006 Zhejiang China; 4 Department of Cancer Medicine, Hammersmith Hospital, Imperial College Healthcare NHS Trust, London, W12 0HS UK; 5 Department of Traditional Chinese Medicine, The First Affiliated Hospital of Zhejiang Chinese Medical University, Hangzhou, 310006 Zhejiang China

**Keywords:** Outcomes research, Cancer immunotherapy

## Abstract

Anti-PD-1/PD-L1 inhibitors provide a survival advantage over conventional therapies for treatment of advanced or metastatic cancer. However, the factors determining which patients benefit the most from anti-PD-1/PD-L1 inhibitors are unknown, making treatment-related decisions difficult. We performed a systematic review and meta-analysis of acquired data to assess the efficacy and toxicity of anti-PD-1/PD-L1 inhibitors in advanced and metastatic cancer. A thorough search strategy was applied to identify randomised controlled trials (RCTs) in Pubmed, Embase, Cochrane, and major conferences. Studies meeting predefined selection criteria were selected, and two independent investigators performed data extraction; overall survival (OS), progression-free survival (PFS), and overall response rate were compared between anti-PD-1/PD-L1 inhibitors and control therapies. We calculated the pooled response rate and 95% CIs of all-grade and high-grade (≥3) adverse effects and evaluated the within-study heterogeneity using subgroup, sensitivity, and meta-regression analyses. In final, we included eligible 35 RCTs (21047 patients). The main estimated hazard ratios (HRs) for OS and PFS were 0.76 (0.71–0.82) and 0.81 (0.73–0.89) in a random-effects model. The anti-PD-1/PD-L1 inhibitor group had a significantly high risk for all-grade immune-related adverse events. Anti-PD-1/PD-L1 inhibitors were identified as a preferable treatment option for advanced or metastatic cancer patients who are male, aged < 65 years, current or former smokers, had no CNS or liver metastasis, had not EGFR mutation, and had high PD-L1 expression.

## Introduction

Cancer is a common cause of death, accounting for more than 9.56 million deaths annually^1^. Over half of cancer patients have a poor prognosis due to locally advanced or systemic metastasis. For the majority of these cases, treatment with conventional therapies, such as chemotherapy and radiotherapy, does not improve their prognosis. Recently, several immune checkpoint inhibitors (ICIs), have been developed and approved for a wide range of tumour types and having shown potential for maintaining homeostasis and eliminating tumour cells. Immunotherapies targeting immune checkpoint pathways have shown potential for generating a durable response and for prolonging disease stabilisation in a significant proportion of inoperable, advanced, or recurrent cancers in patients with multiple cancer types, along with favourable tolerability. In addition to their use as a monotherapy, the general safety of immune checkpoint agents also allows for their use in the development of combined therapies for cancer treatment; combining ICIs with other conventional treatments or targeted therapies is expected to improve anti-tumour activity and increase ICI efficacy. However, although durable responses were reported in cancer patients treated with combination strategies involving ICIs, it is still necessary to optimise dose selection to minimise the adverse events (AEs) caused by combination regimens while maintaining stable clinical effectiveness^2^. Recent research on the anti-tumour activity of the immune system has resulted in the application of immunotherapy as the leading treatment strategy in advanced or metastatic cancer^3^.

Based on published clinical trials, we know that ICIs targeting the PD-1/PD-L1 pathway have a beneficial effect on the treatment of patients with advanced or metastatic cancer compared to conventional therapy^4^. However, the specific factors that help determine which patients would benefit the most from anti-PD-1/PD-L1 inhibitors remain unclear, which in turn can make treatment-related decisions difficult. A previous meta-analysis reported that PD-1/PD-L1 inhibitors are better tolerated than conventional chemotherapy, but they did not analyse survival benefit and tumour response^5^. Another meta-analysis^6^ suggested that anti-PD-1/PD-L1 inhibitors could improve PFS but not OS in NSCLC when compared with control therapies. Moreover, their data were obtained from indirect comparisons, and they only included EGFR-TKIs as control therapy. Wang *et al*.^7^ demonstrated that ICIs are effective in patients with NSCLC, but this meta-analysis failed to compare different tumour subtypes and focused on anti-PD-1/PD-L1 and anti-CTLA-4 inhibitors concurrently.

Here, we performed a systematic review and meta-analysis to compare the clinical efficacy and immune-related AEs of anti-PD-1/PD-L1 inhibitors to optimise the management of advanced or metastatic cancer. Furthermore, we also focused on the association between anti-PD-1/PD-L1 inhibitors and potential subgroup differences, such as ICI monotherapy or ICI-based combined therapy, multiple types of regimens combined with anti-PD-1/PD-L1 inhibitors, and potential biomarkers of immune checkpoint blockade therapy.

## Results

### Literature search and eligible studies

**Figure 1:**
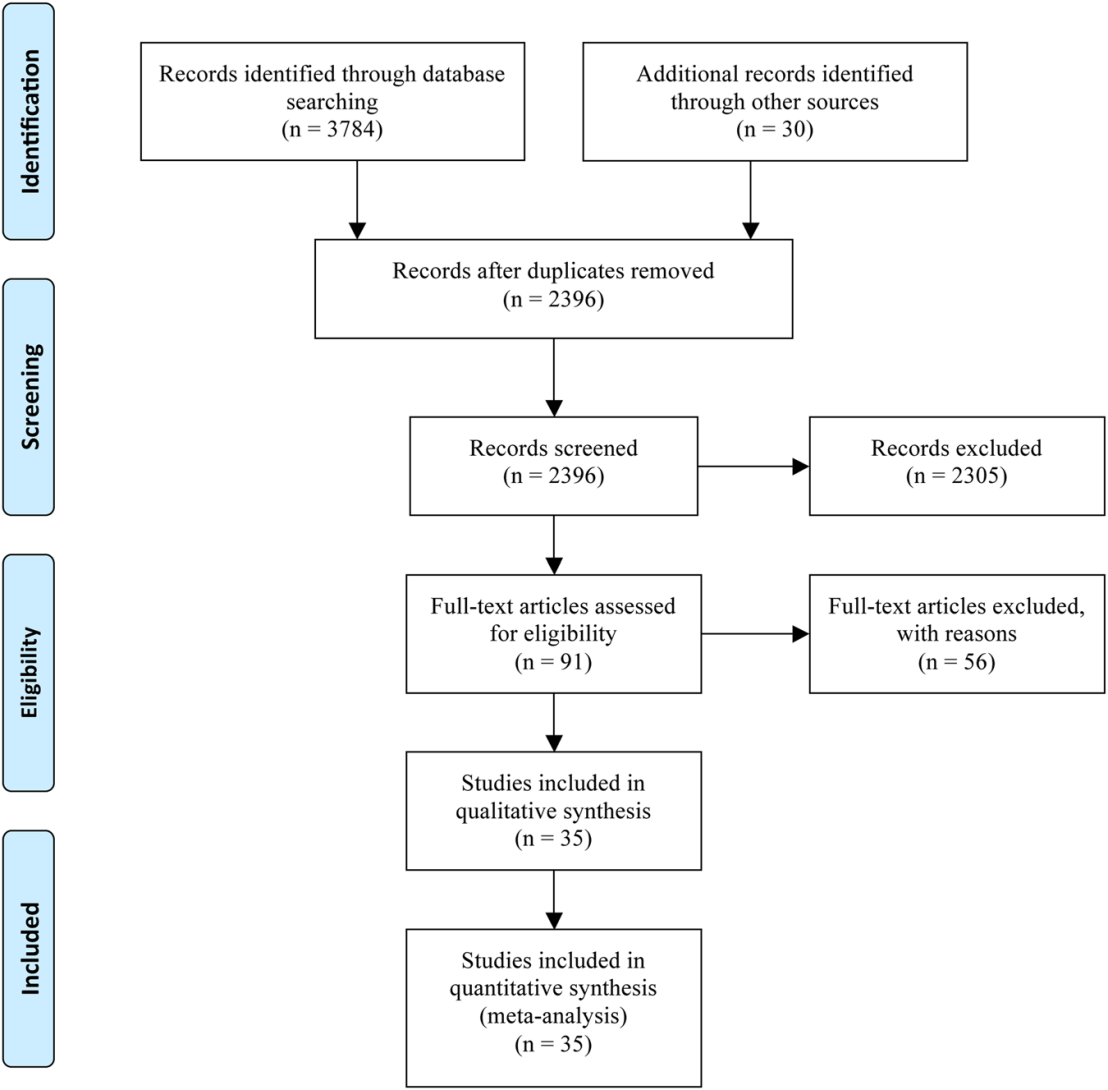
Articles retrieved and assessed for eligibility. After screening, 35 RCTs met the inclusion criteria and were included in the final analysis.

After removal of 1418 duplications following the initial selection, 2396 relevant articles in total were electronically retrieved, from which 91 articles met the prespecified inclusion criteria. After an initial review of the titles and abstracts, 2305 studies were excluded since they failed to meet the following criteria: not randomised controlled trials (RCTs), not reviews, not using anti-PD-1/PD-L1 inhibitors, systematic reviews and meta-analysis, case report, conference reports, only abstract, using anti-PD-1/PD-L1 inhibitors in the control group, using anti-CTLA-4 inhibitors in the intervention group, and single-arm study design. We then screened the remaining 91 articles by assessing the full text and identified 35 RCTs (Horn^8^, Cohen^9^, Schmid^10^, Powles^11^, Paz-Ares^12^, Larkin^13^, Herbst^14^, Gandhi^15^, Fehrenbacher^16^, Barlesi^17^, Antonia^18^, Reck^19^, Kang^20^, Carbone^21^, Bellmunt^22^, Borghaei^23^, Brahmer^24^, Motzer^25^, Fehrenbacher^26^, Ferris^27^, Socinski^28^, Ascierto^29^, Bang^30^, Mateos^31^, Usmani^32^, Borghaei^33^, Eng^34^, Fradet^35^, Hamid^36^, Mok^37^, Motzer^38^, Weber^39^, West^40^, Brian^41^, Rini^42^) that analysed the efficacy and toxicity of anti-PD-1/PD-L1 inhibitors. The selection steps are summarised in the flow diagram in Fig. 1.

### Characteristics of included trials and patients

All trials were performed in advanced or metastatic settings, and sixteen trials involved patients with lung cancer while nineteen involved those with other types of tumours. Fifteen trials were performed with first-line therapy, while the remaining were performed with subsequent-line therapy. One phase II/III, three phase II, and all phase III RCTs were considered eligible for the meta-analysis. Of the included RCTs, twenty-two trials focused on anti-PD-1 inhibitors and thirteen on anti-PD-L1 inhibitors. Anti-PD-1/PD-L1 inhibitors were compared with chemotherapy alone in nineteen trials and with targeted drug therapy in seven trials. In twenty-three trials, the anti-PD-1/PD-L1 inhibitor alone group was compared with the control group, and in thirteen trials, the anti-PD-1/PD-L1 inhibitors plus other drugs group was compared with the control group.

A total of 21047 patients from 35 RCTs were enrolled in our meta-analysis, and 10248 (48.7%) had advanced or metastatic lung cancer, while the remaining had melanoma (8.4%), head-and-neck cancer (4.1%), gastric or gastro-oesophageal junction cancer (4.1%), renal-cell carcinoma (16.5%), multiple myeloma (2.6%), urothelial carcinoma (9.6%), breast cancer (4.3%) and colorectal cancer (1.7%). The age of participants ranged from 15 to 90 years across all studies. The median follow-up period ranged from 5.1 to 38.5 months. The main characteristics and results of each trial are presented in Table 1 and Supplementary Table 1.

**Table 1:** Characteristics of the studies included in this meta-analysis.

Author, year	Histology	Masking	Line	Treatment arm	HR (95% CI) for PFS	HR (95% CI) for OS
Horn 2018	SCLC	Double-Blind	1	Atezolizumab + CE vs Placebo + CE	0.77 (0.62–0.96)	0.70 (0.54–0.91)
Cohen 2018	HNC	Open-Label	>1	Pembrolizumab vs IC of Standard-of-care Therapy	0.96 (0.79–1.16)	0.80 (0.65–0.98)
Schmid 2018	BC	Double-Blind	1	Atezolizumab + Nab-Paclitaxel vs Placebo + Nab-Paclitaxel	0.80 (0.69–0.92)	0.84 (0.69–1.02)
Powles 2018	UC	Open-Label	>1	Atezolizumab vs ICC	NA	0.85 (0.73–0.99)
Paz-Ares 2018	NSCLC	Double-Blind	1	Pembrolizumab + ICC vs Placebo + ICC	0.56 (0.45–0.70)	0.64 (0.49–0.85)
Larkin 2018	Mm	Open-Label	>1	Nivolumab vs ICC	1.03 (0.78–1.44)	0.95 (0.73–1.24)
Herbst 2016	NSCLC	Open-Label	>1	Pembrolizumab 2 mg/kg vs Docetaxel	0.88 (0.74–1.05)	0.71 (0.58–0.88)
				Pembrolizumab 10 mg/kg vs Docetaxel	0.79 (0.66–0.94)	0.61 (0.49–0.75)
Gandhi 2018	NSCLC	Double-Blind	1	Pembrolizumab + IC of PT-DC vs Placebo + IC of PT-DC	0.52 (0.43–0.64)	0.49 (0.38–0.64)
Fehrenbacher 2018	NSCLC	Open-Label	>1	Atezolizumab vs Docetaxel	0.96 (0.85–1.08)	0.80 (0.70–0.92)
Barlesi 2018	NSCLC	Open-Label	>1	Avelumab vs Docetaxel	1.16 (0.97–1.40)	0.90 (0.75–1.08)
Antonia 2018	NSCLC	Double-Blind	>1	Durvalumab vs Placebo	0.51 (0.41–0.63)	0.68 (0.47–0.997)
Reck 2016	NSCLC	Open-Label	1	Pembrolizumab vs IC of PT-DC	0.50 (0.37–0.68)	0.60 (0.41–0.89)
Kang 2017	GC/GEJC	Double-Blind	>1	Nivolumab vs Placebo	0.60 (0.49–0.75)	0.63 (0.51–0.78)
Carbone 2017	NSCLC	Open-Label	1	Nivolumab vs IC of PT-DC	1.19 (0.97–1.46)	1.08 (0.87–1.34)
Bellmunt 2017	UC	Open-Label	>1	Pembrolizumab vs ICC	0.98 (0.81–1.19)	0.73 (0.59–0.91)
Borghaei 2015	NSCLC	Open-Label	>1	Nivolumab vs Docetaxel	0.92 (0.77–1.11)	0.73 (0.59–0.89)
Brahmer 2015	NSCLC	Open-Label	>1	Nivolumab vs Docetaxel	0.62 (0.47–0.81)	0.59 (0.44–0.79)
Motzer 2015	RCC	Open-Label	>1	Nivolumab vs Everolimus	0.88 (0.75–1.03)	0.73 (0.57–0.93)
Fehrenbacher 2016	NSCLC	Open-Label	>1	Atezolizumab vs Docetaxel	0.94 (0.72–1.23)	0.73 (0.53–0.99)
Ferris 2016	HNC	Open-Label	>1	Nivolumab vs IC of Standard, Single-agent Therapy	0.89 (0.70–1.13)	0.70 (0.51–0.96)
Socinski 2018	NSCLC	Open-Label	1	Atezolizumab + Bev + PC vs Bev + PC	0.61 (0.52–0.72)	NA
Ascierto 2018	Mm	Double-Blind	1	Nivolumab vs Dacarbazine	0.42 (0.33–0.53)	0.46 (0.36–0.59)
Bang 2018	GC/GEJC	Open-Label	>1	Atezolizumab vs ICC	1.73 (1.40–2.20)	1.10 (0.90–1.40)
Mateos 2019	MM	Open-Label	>1	Pembrolizumab plus PD vs PD	1.53 (1.05–2.22)	1.61 (0.91–2.85)
Usmani 2019	MM	Open-Label	1	Pembrolizumab plus LD vs LD	1.22 (0.67–2.22)	2.06 (0.93–4.55)
Borghaei 2019	NSCLC	Open-Label	1	Pembrolizumab plus Pemetrexed-Carboplatin vs Pemetrexed-Carboplatin	0.53 (0.33–0.86)	0.56 (0.32–0.95)
Eng 2019	CRC	Open-Label	>1	Atezolizumab plus Cobimetinib vs Regorafenib	1.25 (0.94–1.65)	1.00 (0.73–1.38)
				Atezolizumab vs Regorafenib	1.39 (1.00–1.94)	1.19 (0.83–1.71)
Fradet 2019	UC	Open-Label	>1	Pembrolizumab vs ICC	0.96 (0.79–1.16)	0.70 (0.57–0.85)
Hamid 2019	Mm	Double-Blind	>1	Pembrolizumab 2 mg/kg vs ICC	0.58 (0.46–0.73)	0.79 (0.58–1.08)
				Pembrolizumab 10 mg/kg vs ICC	0.47 (0.37–0.60)	0.67 (0.49–0.92)
Mok 2019	NSCLC	Open-Label	1	Pembrolizumab vs IC of PT-DC	1.07 (0.94–1.21)	0.81 (0.71–0.93)
Motzer 2019	RCC	Open-Label	1	Avelumab plus Axitinib vs Sunitinib	0.69 (0.56–0.84)	0.78 (0.55–1.08)
Weber 2015	Mm	Open-Label	>1	Nivolumab vs ICC	0.82 (0.32–2.05)	NA
West 2019	NSCLC	Open-Label	1	Atezolizumab plus IC of PT-DC vs IC of PT-DC	0.65 (0.54–0.77)	0.80 (0.65–0.99)
Brian 2019	RCC	Open-Label	1	Atezolizumab plus Bev vs Sunitinib	0.83 (0.70–0.97)	0.93 (0.76–1.14)
Rini 2019	RCC	Open-Label	1	Pembrolizumab plus Axitinib vs Sunitinib	0.69 (0.57–0.84)	0.53 (0.38–0.74)

Abbreviations: NSCLC, Non-Small Cell Lung Cancer; SCLC, Small Cell Lung Cancer; Mm, Melanoma; RCC, Renal-Cell Carcinoma; UC, Urothelial Carcinoma; GC/GEJC, Gastric or Gastro-oesophageal Junction Cancer; BC, Breast Cancer; HNC, Head-and-Neck Cancer, MM, Multiple Myeloma. CE, Carboplatin plus Etoposide; IC, Investigator’s Choice; ICC = Investigator’s Choice of Chemotherapy; PT-DC, Platinum-Based Doublet Chemotherapy; PC, Paclitaxel plus Carboplatin; Bev, Bevacizumab; Pomalidomide plus Dexamethasone, PD; Pembrolizumab plus Lenalidomide and Dexamethasone, LD. Lenalidomide and Dexamethasone.

### Assessment of methodological bias

The random sequence was generated using an interactive voice or web response system in eighteen studies (Bang 2018; Barlesi 2018; Brian 2019; Cohen 2018; Eng 2019; Fehrenbacher 2016; Gandhi 2018; Herbst 2016; Horn 2018; Kang 2017; Mateos 2019; Mok 2019; Paz-Ares 2018; Powles 2018; Schmid 2018; Usmani 2019; Weber 2015; West 2019). Except for those eleven trials (Bang 2018; Borghaei 2015; Borghaei 2019; Brahmer 2015; Carbone 2017; Fradet 2019; Larkin 2018; Motzer 2015; Motzer 2019; Rini 2019; Socinski 2018), the remaining studies failed to provide information that helped us to assess allocation concealment. In addition to the study by Antonia 2018, Ascierto 2018, Gandhi 2018, Hamid 2017, Horn 2018, Kang 2018, Paz-Ares 2018, Powles 2018, Schmid 2018, other studies failed to contain detailed information about blinding of the participants and personnel. All studies but that by Cohen 2018, Ascierto 2018, Eng 2019, Fehrenbacher 2018, Fradet 2019, Hamid 2017 and Mok 2019 involved the blinding of outcome assessment. There were no obvious selective reporting existing across other studies and ultimately complete report the final outcomes initially defined. Except for twenty-three studies, the remaining studies (Barlesi 2018; Bellmunt 2017; Brahmer 2015; Cohen 2018; Fehrenbacher 2016; Fehrenbacher 2018; Ferris 2016; Gandhi 2018; Herbst 2016; Horn 2018; Reck 2016; Socinski 2018). The assessment of risk of reporting bias, attrition bias, and other bias are listed in Supplementary Figs. 1 and 2. The detailed methods are summarised in Supplementary Method 2^43^.

### Efficacy

**Figure 2:**
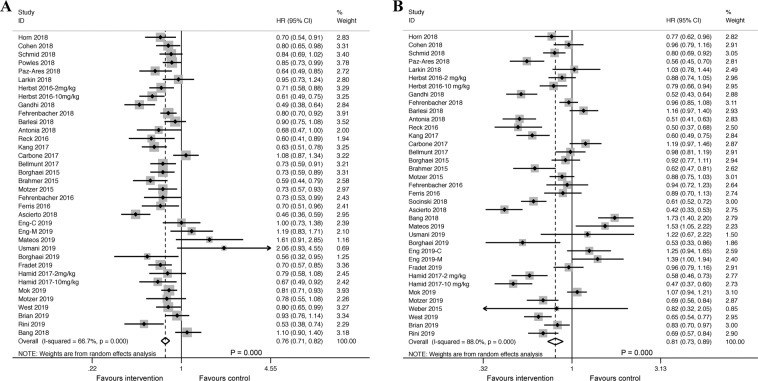
Forest plot of the meta-analysis for estimating the HR and 95% CIs for OS (**A**) and PFS (**B**) in the intervention group, compared with that in the control group (OS: HR: 0.76; 95% CI, 0.71–0.82, *P* = *0*.*000*; PFS: HR: 0.81; 95% CI, 0.73–0.89, *P* = *0*.*000*). Squares indicate study-specific HRs. Horizontal lines crossing the square indicate the 95% CIs. The dashed vertical lines indicate the specific pooled HR. Diamonds indicate the estimated overall effect according to meta-analysis random effect of pooled HRs from all included studies with their corresponding 95% CIs. Favours intervention shows that anti-PD-1/PD-L1 inhibitors (Atezolizumab, Pembrolizumab, Nivolumab, Avelumab or Durvalumab) had survival benefit over the control therapies (placebo, chemotherapy, targeted therapy alone or their combination); Favours control shows that the control therapies had survival benefit over anti-PD-1/PD-L1 inhibitors. Herbst 2016-2 mg/kg, 2 mg/kg subset in Herbst 2016; Herbst 2016-10 mg/kg, 10 mg/kg subset in Herbst 2016; Eng-C 2019, combined therapy subset in Eng 2019; Eng-M 2019, monotherapy subset in Eng 2019; Hamid 2017-2 mg/kg, 2 mg/kg subset in Hamid 2017; Hamid 2017-10 mg/kg, 10 mg/kg subset in Hamid 2017.

Acquired data on OS and PFS were available from 35 trials including 21047 patients. In OS analysis, the pooled hazard ratio (HR) was 0.76 with a 95% confidence interval (CI) of 0.71–0.82 in the intervention group with anti-PD-1/PD-L1 inhibitors (Fig. 2A) when compared with patients in control group. Thus, anti-PD-1/PD-L1 therapies resulted in better OS. Simultaneously, the pooled HR for PFS revealed a significantly lower risk of recurrence and progression with anti-PD-1/PD-L1 therapies in all patients (HR: 0.81; 95% CI, 0.73–0.89) (Fig. 2B). However, moderate to high heterogeneity was observed among trials in the OS (I^2^ = 66.7%, *P* ≤ *0*.*001*) and PFS (I^2^ = 88.0%, *P* ≤ *0*.*001*) subsets, which suggested that the pooled HRs were supposed to be calculated using the random-effects model (Fig. 2).

### PD-L1 expression as a biomarker and predictor of OS and PFS

PD-L1 tumour expression scores were categorised into high and low expression categories using different cut-off values (<1% and ≥1% and <50% and ≥50%) to analyse the correlation of PD-L1 expression and anti-PD-1/PD-L1 inhibitor response.

As shown in Supplementary Figs. 3 and 4, survival benefit from anti-PD-1/PD-L1 inhibitors increased with the up-regulation of PD-L1 expression on tumour cells. Similarly, both OS and PFS improvement were significant with anti-PD-1/PD-L1 therapies in patients with PD-L1 expression of ≥1%, but not in those with PD-L1 expression of <1%. Additionally, a slight improvement in OS was observed with the increase in PD-L1 tumour expression from ≥1% (HR: 0.70; 95% CI, 0.65–0.76) to ≥50% (HR: 0.60; 95% CI, 0.53–0.68) and in PFS from ≥1% (HR: 0.74; 95% CI, 0.65–0.85) to ≥50% (HR: 0.59; 95% CI, 0.49–0.72); however, statistical significance was absent.

### Selected subgroup analysis

Subgroup analysis of OS and PFS were performed across predefined subgroups (Table 2 and Supplementary Tables 2 and 3). Survival benefit was explored across multiple types of cancer, including haematological malignancy, and subgroup analysis suggested that those with non-small cell lung cancer, melanoma, renal-cell carcinoma, and multiple myeloma gained benefit from anti-PD-1/PD-L1 inhibitors in terms of both OS and PFS. Survival benefit was observed in patients with negative EGFR mutation receiving anti-PD-1/PD-L1 inhibitors (OS: HR: 0.70, 95% CI: 0.63–0.77, *P* ≤ *0*.*001*; PFS: HR: 0.83, 95% CI: 0.73–0.95, *P* = *0*.*007*) but not in those with positive EGFR mutation (OS: HR: 1.11, 95% CI: 0.80–1.52, *P* = *0*.*538*; PFS: HR: 1.57, 95% CI: 1.07–2.31; *P* = *0*.*022*). Contrastingly, anti-PD-1/PD-L1 inhibitors failed to achieve a better OS and PFS in both patients with positive (OS: HR: 0.87, 95% CI: 0.63–1.21, *P* = *0*.*408*; PFS: HR: 1.09, 95% CI: 0.82–1.43; *P* = *0*.*563*) and negative RAS mutations (OS: HR: 0.99, 95% CI: 0.81–1.20, *P* = *0*.*900*; PFS: HR: 1.45, 95% CI: 1.14–1.84; *P* = *0*.*002*). Separately, cancer patients without CNS metastasis were likely to gain survival benefits from anti-PD-1/PD-L1 inhibitors (OS: *P* ≤ *0*.*001*; PFS: *P* ≤ *0*.*001*); unlikely, only PFS benefit was observed in cancer patients with CNS metastasis (PFS: *P* = *0*.*036*), but not observed in OS (OS: *P* = *0*.*303*). Additionally, it was also evident that anti-PD-1/PD-L1 inhibitors showed the maximum benefits in current/former smokers (OS: HR: 0.75, 95% CI: 0.68–0.83, *P* ≤ *0*.*001*; PFS: HR: 0.72, 95% CI: 0.57–0.89, *P* = *0*.*003*), rather than in never smoker (OS: HR: 0.81, 95% CI: 0.66–1.00, *P* = *0*.*045*; PFS: HR: 1.00, 95% CI: 0.55–1.79, *P* = *0*.*991*). Furthermore, anti-PD-1/PD-L1 inhibitors provided better survival benefits in younger patients with a age cut-off for OS (75 yr: *P* ≤ *0*.*001* vs *P* = *0*.*616*), but not for PFS of all patients. Meanwhile, on considering double-blind status, anti-PD-1/PD-L1 inhibitors showed a PFS advantage compared with control therapies (*P* ≤ *0*.*001*), but no such significant difference was found in open-label studies (*P* = *0*.*054*). Unlikely, in OS analysis, that offer roughly the same outcomes between double-blind trials (HR: 0.64, 95% CI: 0.56–0.74) and open-label trials (HR: 0.81, 95% CI: 0.75–0.87) for which HR was almost similar. Interestingly, anti-PD-1/PD-L1 inhibitor therapy was related to benefits in PFS in men (PFS: *P* = *0*.*002*) but not in women (PFS: *P* = *0*.*196*); however, OS benefit was both observed in male (*P* ≤ *0*.*001*) and female (*P* ≤ *0*.*001*). In the OS analysis, anti-PD-1/PD-L1 inhibitors both provided survival benefits when ICIs used as first-line treatment (HR: 0.72, 95% CI: 0.62–0.84) and subsequent-line setting (HR: 0.78, 95% CI: 0.73–0.85) with significant difference. Conversely, in PFS analysis, survival improvement was evident in the first-line setting (HR: 0.70, 95% CI: 0.60–0.81) but not in the subsequent-line setting (HR: 0.89, 95% CI: 0.79–1.01). Although anti-PD-1/PD-L1 inhibitors increased the PFS and OS irrespective of the regimen used, HRs of survival favoured anti-PD-1/PD-L1 inhibitors in combination with chemotherapy over control therapies (OS: *P* ≤ *0*.*001*; PFS: *P* ≤ *0*.*001*) but not when they were combined with targeted therapy (OS: *P* = *0*.*694*; PFS: *P* = 602) in combination therapy. The survival benefit from anti-PD-1/PD-L1 inhibitors varied between the two targets in PFS analysis (PD-L1: 0.88, 95% CI: 0.75–1.04, *P* = *0*.*143*; PD-1: 0.77, 95% CI: 0.68–0.87, *P* ≤ *0*.*001*) but not in OS analysis (PD-L1: 0.86, 95% CI: 0.80–0.92, *P* ≤ *0*.*001*; PD-1: 0.71, 95% CI: 0.64–0.78, *P* ≤ *0*.*001*). Additionally, in both OS and PFS analyses, patients gained survival benefits in the pembrolizumab and nivolumab subsets, but not in the avelumab subset (OS: *P* = *0*.*484*; PFS: *P* = *0*.*675*). OS-related benefits were not only significant in patients without liver metastasis for OS (*P* ≤ *0*.*001*), but also observed in those with liver metastasis (*P* = *0*.*017*). Moreover, there was an evident trend to favour ICIs than control therapies for cancer patients in control group with placebo (OS: HR: 0.64, 95% CI: 0.53–0.77, *P* ≤ *0*.*001*; PFS: HR: 0.55, 95% CI: 0.47–0.65; *P* ≤ *0*.*001*) and chemotherapy (OS: HR: 0.74, 95% CI: 0.68–0.80, *P* ≤ *0*.*001*; PFS: HR: 0.79, 95% CI: 0.69–0.90; *P* ≤ *0*.*001*), but not with biologics (OS: HR: 0.93, 95% CI: 0.74–1.16, *P* = *0*.*496* PFS: HR: 0.97, 95% CI: 0.80–1.17; *P* = *0*.*730*). In the subgroup analysis for ECOG PS 0 and 1, the HRs for OS were 0.81 (95% CI: 0.71–0.93) and 0.74 (95% CI: 0.68–0.80), respectively, while the HRs for PFS were 0.91 (95% CI: 0.67–1.22) and 0.77 (95% CI: 0.64–0.93), respectively.

**Table 2:** Selected subgroup analysis of survival in the intent-to-treat population.

Variables		OS		PFS	
		HR (95% CI)	*P*	HR (95% CI)	*P*
Histotype	HNC	0.77 (0.65, 0.91)	0.003	0.93 (0.80, 1.08)	0.357
	GC/GEJC	0.83 (0.48, 1.44)	0.509	1.02 (0.36, 2.87)	0.973
	UC	0.77 (0.68, 0.87)	≤0.001	0.97 (0.85, 1.11)	0.660
	NSCLC	0.72 (0.66, 0.80)	≤0.001	0.75 (0.65, 0.87)	≤0.001
	MM	1.75 (1.10, 2.78)	0.018	1.44 (1.05, 1.97)	0.026
	Mm	0.69 (0.50, 0.96)	0.029	0.60 (0.43, 0.83)	0.002
	RCC	0.74 (0.59, 0.93)	0.010	0.78 (0.69, 0.88)	≤0.001
	CRC	1.08 (0.85, 1.37)	0.533	1.31 (1.05, 1.62)	0.014
Regimen	Combination therapy	0.78 (0.66, 0.91)	0.002	0.75 (0.65, 0.86)	≤0.001
	Monotherapy	0.76 (0.70, 0.82)	≤0.001	0.84 (0.74, 0.95)	0.007
Combination Drug	Chemotherapy	0.68 (0.57, 0.81)	≤0.001	0.64 (0.55, 0.76)	≤0.001
	Targeted therapy	0.94 (0.71, 1.26)	0.694	0.94 (0.74, 1.19)	0.602
Treatment in control group	Chemotherapy	0.74 (0.68, 0.80)	≤0.001	0.79 (0.69, 0.90)	≤0.001
	Placebo	0.64 (0.53, 0.77)	≤0.001	0.55 (0.47, 0.65)	≤0.001
	Biologics	0.93 (0.74, 1.16)	0.496	0.97 (0.80, 1.17)	0.730
Age	<65yr	0.77 (0.69, 0.87)	≤0.001	0.82 (0.67, 1.02)	0.078
	≥65yr	0.76 (0.67, 0.84)	≤0.001	0.84 (0.69, 1.01)	0.067
	≥65 to <75yr	0.64 (0.54, 0.77)	≤0.001	0.71 (0.39, 1.29)	0.264
	≥75yr	0.88 (0.53, 1.46)	0.616	0.98 (0.52, 1.85)	0.956
Sex	Male	0.74 (0.69, 0.79)	≤0.001	0.75 (0.63, 0.90)	0.002
	Female	0.75 (0.63, 0.88)	≤0.001	0.82 (0.61, 1.11)	0.196
ECOG	0	0.81 (0.71, 0.93)	0.002	0.91 (0.67, 1.22)	0.530
	1	0.74 (0.68, 0.80)	≤0.001	0.77 (0.64, 0.93)	0.005
Smoking	Current/Former	0.75 (0.68, 0.83)	≤0.001	0.72 (0.57, 0.89)	0.003
	Never	0.81 (0.66, 1.00)	0.045	1.00 (0.55, 1.79)	0.991
Line	First-line	0.72 (0.62, 0.84)	≤0.001	0.70 (0.60, 0.81)	≤0.001
	Subsequent line	0.78 (0.73, 0.85)	≤0.001	0.89 (0.79, 1.01)	0.081
Masking	Double-blind	0.64 (0.56, 0.74)	≤0.001	0.57 (0.49, 0.67)	≤0.001
	Open-label	0.81 (0.75, 0.87)	≤0.001	0.91 (0.82, 1.00)	0.054
Target spot	PD-L1	0.86 (0.80, 0.92)	≤0.001	0.88 (0.75, 1.04)	0.143
	PD-1	0.71 (0.64, 0.78)	≤0.001	0.77 (0.68, 0.87)	≤0.001
Anti-PD-1/PD- L1 inhibitor used	Atezolizumab	0.84 (0.78, 0.91)	≤0.001	0.86 (0.74, 1.00)	0.045
	Pembrolizumab	0.70 (0.63, 0.79)	≤0.001	0.76 (0.65, 0.89)	≤0.001
	Nivolumab	0.71 (0.59, 0.86)	≤0.001	0.78 (0.63, 0.98)	0.029
	Avelumab	0.94 (0.79, 1.12)	0.484	1.11 (0.68, 1.83)	0.675
CNS Metastasis	Yes	0.78 (0.48, 1.25)	0.303	0.64 (0.42, 0.97)	0.036
	No	0.70 (0.61, 0.80)	≤0.001	0.66 (0.52, 0.83)	≤0.001
Liver Metastasis	Yes	0.81 (0.68, 0.96)	0.017	—	—
	No	0.71 (0.62, 0.82)	≤0.001	—	—
RAS	Mutant	0.87 (0.63, 1.21)	0.408	1.09 (0.82, 1.43)	0.563
	Wildtype	0.99 (0.81, 1.20)	0.900	1.45 (1.14, 1.84)	0.002
EGFR	Mutant	1.11 (0.80, 1.52)	0.538	1.57 (1.07, 2.31)	0.022
	Wildtype	0.70 (0.63, 0.77)	≤0.001	0.83 (0.73, 0.95)	0.007

Abbreviations: NSCLC, Non-Small Cell Lung Cancer; SCLC, Small Cell Lung Cancer; Mm, Melanoma; RCC, Renal-Cell Carcinoma; UC, Urothelial Carcinoma; GC/GEJC, Gastric or Gastro-oesophageal Junction Cancer; BC, Breast Cancer; HNC, Head-and-Neck Cancer, MM, Multiple Myeloma.

### Overall response rate

**Figure 3:**
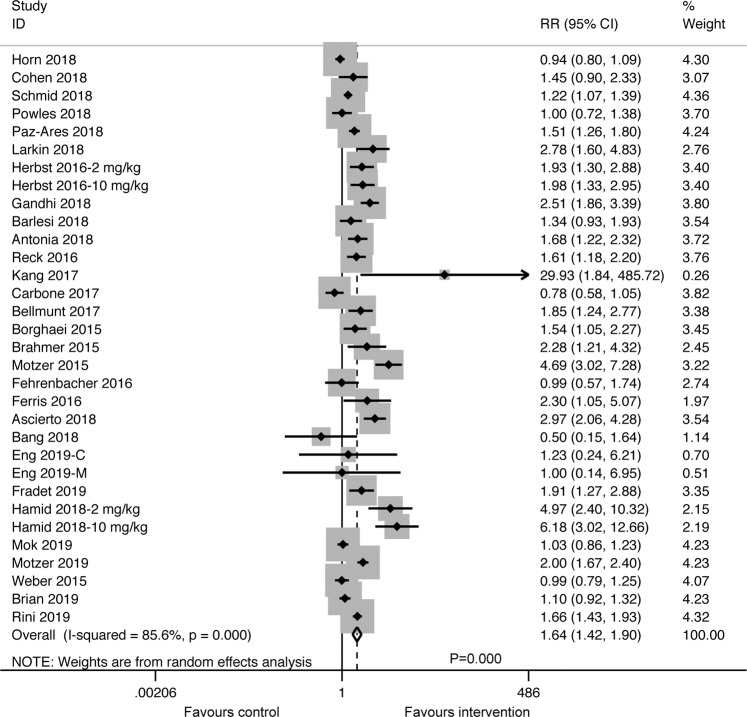
Forest plot of the meta-analysis for estimating the RR and 95% CIs for the ORR in the intervention group, compared with that in the control group (RR: 1.64; 95% CI, 1.42–1.90, *P* = *0*.*000*). Squares indicate study-specific RRs. Horizontal lines crossing the square indicate the 95% CIs. The dashed vertical lines indicate the specific pooled RR. Diamonds indicate the estimated overall effect according to meta-analysis random effect of pooled RRs from all included studies with their corresponding 95% CIs. Favours intervention represents better complete response or partial response in the treatment of anti-PD-1/PD-L1 inhibitors (Atezolizumab, Pembrolizumab, Nivolumab, Avelumab or Durvalumab); Favours control represents better complete response or partial response in the treatment of the control therapies (placebo, chemotherapy, targeted therapy alone or their combination). Herbst 2016-2 mg/kg, 2 mg/kg subset in Herbst 2016; Herbst 2016-10 mg/kg, 10 mg/kg subset in Herbst 2016; Eng-C 2019, combined therapy subset in Eng 2019; Eng-M 2019, monotherapy subset in Eng 2019; Hamid 2017-2 mg/kg, 2 mg/kg subset in Hamid 2017; Hamid 2017-10 mg/kg, 10 mg/kg subset in Hamid 2017.

Thirty-three RCTs providing valid data for ORR revealed that cancer patients gained a significant ORR benefit from anti-PD-1/PD-L1 inhibitors (RR:1.64; 95% CI: 1.42–1.90; *P* ≤ *0*.*001*) (Fig. 3). And similar to survival analysis, better ORR was observed with anti-PD-1 (RR: 1.94; 95% CI: 1.59–2.37; *P* ≤ *0*.*001*) inhibitors, when compared to anti-PD-L1 (RR: 1.22; 95% CI: 1.00–1.49; *P* = *0*.*046*) inhibitors. In terms of individual agents, better overall response was reported for nivolumab (RR: 2.07; 95% CI: 1.29–3.33; *P* = *0*.*003*) and pembrolizumab (RR: 1.88; 95% CI: 1.53–2.31; *P* ≤ *0*.*001*), while no obvious improvement was found with atezolizumab (RR: 1.08; 95% CI: 0.98–1.19; *P* = *0*.*133*) and avelumab (RR: 1.43; 95% CI: 0.88–2.34; *P* = *0*.*151*) (Supplementary Fig. 5A,B). Meta-regression indicated that several of the examined factors were potentially responsible for between-study heterogeneity in ORR, including target spot (*P* ≤ *0*.*001*) and regimen (*P* ≤ *0*.*001*) (Supplementary Fig. 6A,B).

### Safety

Anti-PD-1/PD-L1 inhibitor therapy resulted in a significantly higher risk for all-grade immune-related AEs (Table 3). Patients treated with anti-PD-1/PD-L1 inhibitors had a slightly lower RR of AEs such as anaemia (*P* ≤ *0*.*001*), neutropenia (*P* ≤ *0*.*001*), diarrhoea (*P* = *0*.*020*), stomatitis (*P* ≤ *0*.*001*), nausea (*P* ≤ *0*.*001*), asthenia (*P* ≤ *0*.*001*), alopecia (*P* ≤ *0*.*001*), neuropathy (*P* ≤ *0*.*001*), fatigue (*P* ≤ *0*.*001*), vomit (*P* ≤ *0*.*001*), constipation (*P* ≤ *0*.*001*), mucosal inflammation (*P* ≤ *0*.*001*), decreased neutrophil count (*P* ≤ *0*.*001*), decreased appetite (*P* ≤ *0*.*001*), and decreased white-cell count (*P* ≤ *0*.*001*). Conversely, the risk of all-grade pneumonitis (*P* = *0*.*011*), pyrexia (*P* = *0*.*024*), rash (*P* ≤ *0*.*001*), and pruritus (*P* ≤ *0*.*001*) were higher in patients treated with anti-PD-1/PD-L1 inhibitors than in those receiving control therapies. Only the risk of arthralgia (*P* = *0*.*728*) and dyspnoea (*P* = *0*.*943*) did not differ significantly.

**Table 3:** Treatment-related common adverse events in this meta-analysis.

Adverse events	All grades		Grade ≥ 3	
	RR (95% CI)	*P*	RR (95% CI)	*P*
Anemia	0.35 (0.27, 0.46)	≤0.001	0.34 (0.23, 0.49)	≤0.001
Alopecia	0.12 (0.08, 0.19)	≤0.001	0.29 (0.11, 0.76)	0.012
Arthralgia	1.05 (0.80, 1.38)	0.728	1.49 (0.79, 2.84)	0.219
Neutropenia	0.16 (0.11, 0.25)	≤0.001	0.22 (0.14, 0.34)	≤0.001
Diarrhea	0.82 (0.70, 0.97)	0.020	0.97 (0.68, 1.38)	0.859
Dyspnea	0.99 (0.84, 1.17)	0.943	0.95 (0.65, 1.38)	0.790
Stomatitis	0.31 (0.20, 0.48)	≤0.001	0.31 (0.14, 0.70)	0.005
Nausea	0.60 (0.49, 0.73)	≤0.001	0.76 (0.54, 1.05)	0.098
Pyrexia	1.23 (1.03, 1.48)	0.024	0.92 (0.40, 2.10)	0.844
Asthenia	0.69 (0.60, 0.79)	≤0.001	0.55 (0.39, 0.79)	≤0.001
Rash	1.56 (1.26, 1.92)	≤0.001	1.31 (0.80, 2.15)	0.277
Neuropathy	0.39 (0.26, 0.60)	≤0.001	0.81 (0.44, 1.49)	0.504
Fatigue	0.77 (0.70, 0.85)	≤0.001	0.55 (0.42, 0.72)	≤0.001
Vomit	0.61 (0.45, 0.81)	≤0.001	0.66 (0.44, 0.98)	0.040
Constipation	0.63 (0.48, 0.83)	≤0.001	0.78 (0.32, 1.88)	0.575
Pneumonitis	2.44 (1.23, 4.87)	0.011	1.67 (0.94, 2.96)	0.080
Pruritus	3.46 (2.67, 4.49)	≤0.001	0.90 (0.27, 3.00)	0.869
Mucosal Inflammation	0.29 (0.18, 0.44)	≤0.001	0.34 (0.18, 0.66)	0.002
Decreased Appetite	0.19 (0.08, 0.42)	≤0.001	0.76 (0.49, 1.19)	0.228
Decreased Neutrophil Count	0.17 (0.09, 0.22)	≤0.001	0.17 (0.09, 0.35)	≤0.001
Decreased White-Cell Count	0.19 (0.08, 0.42)	≤0.001	0.19 (0.08, 0.49)	≤0.001

Furthermore, to identify the severity of AEs, we examined the risk of high-grade (≥3) AEs. Anti-PD-1/PD-L1 inhibitors resulted in a lower RR of AEs for anaemia (*P* ≤ *0*.*001*), neutropenia (*P* ≤ *0*.*001*), stomatitis (*P* = *0*.*005*), asthenia (*P* ≤ *0*.*001*), vomiting (*P* = *0*.*040*), alopecia (*P* = *0*.*012*), mucosal inflammation (*P* = *0*.*002*), decreased white-cell count (*P* ≤ *0*.*001*), decreased neutrophil count (*P* ≤ *0*.*001*), and fatigue (*P* ≤ *0*.*001*). However, patients receiving anti-PD-1/PD-L1 inhibitors reported a high risk of high-grade (≥3) AEs (without statistical significance) including arthralgia (*P* = *0*.*219*), constipation (*P* = *0*.*575*), dyspnoea (*P* = *0*.*790*), pruritus (*P* = *0*.*869*), pneumonitis (*P* = *0*.*080*), neuropathy (*P* = *0*.*504*), diarrhoea (*P* = *0*.*859*), pyrexia (*P* = *0*.*844*), rash (*P* = *0*.*277*), decreased appetite (*P* = *0*.*228*), and nausea (*P* = *0*.*098*).

### Sensitivity analysis and publication bias

We performed sensitivity analysis to estimate the influence of a single study on the overall results of the meta-analysis (Supplementary Fig. 7A,B). The pooled results were not significantly changed after deleting each trial, which confirmed the rationality and reliability of our meta-analysis.

The shape of the funnel plot generated from the main outcomes revealed moderate asymmetry, which indicates that the possibility of publication bias cannot be ruled out across all studies (Supplementary Fig. 8A,B). Interestingly, both Egger’s (*P* = *0*.*766*; Supplementary Fig. 9A) and Begg’s test (*P* = *0*.*540*; Supplementary Fig. 10A) ruled out publication bias in the OS analysis, which means that there is no significant influence of publication bias. Similarly, neither the Egger’s test (*P* = *0*.*565*; Supplementary Fig. 9B) nor the Begg’s test (*P* = *0*.*685*; Supplementary Fig. 10B) showed an obvious significant association between the study effects and study size in PFS analysis.

## Discussion

Treatment with anti-PD-1/PD-L1 inhibitors is the preferred approach for patients with advanced or metastatic cancer. After examining individual data from 35 RCTs, we revealed that anti-PD-1/PD-L1 inhibitors conferred better PFS and OS and showed better safety than did conventional therapy or placebo. One of clinical trials from Carbone and colleagues^21^ showed no benefit related to OS or PFS. This could be because in their study, nearly 60% of the patients in the control group had also received nivolumab, which was likely to have reversed the effects on OS and PFS. Similarly, the lack of a PFS benefit with anti-PD-1/PD-L1 inhibitors was also observed in nearly half studues including Cohen 2018, Larkin 2018, Herbst 2016, Fehrenbacher 2018, Bellmunt 2017, Borghaei 2015, Motzer 2015, Fehrenbacher 2016, Ferris 2016, Barlesi 2018, Eng 2019, Fradet 2019, Bang 2018, Usmani 2019, Mok 2019, Weber 2 015 and Mateos 2019. Those trials were performed in the subsequent-line setting for previously treated patients.

Moderate to high heterogeneity was observed across studies for OS and PFS. Thus, a random-effect model was applied in our meta-analysis. This substantial heterogeneity could be explained by the broad number of tumour histotypes, masking methods, lines of treatment, and regimens. It is also likely that other factors, such as sex, age, smoking status, PD-L1 expression, CNS or liver metastasis, and *EGFR* or *RAS* mutation, influenced the efficacy of anti-PD-1/PD-L1 inhibitors in all trials. To strengthen our study, we also carried out subgroup, meta-regression, and sensitivity analyses.

PD-L1 expression in tumour cells must be considered when anti-PD-1/PD-L1 inhibitors are used to block the PD-1/PD-L1 pathway. It has been shown that PD-L1 expression is linked with poor prognosis, and thus, responses to anti-PD-1/PD-L1 inhibitors are worth assessing. PD-L1 expression has been identified as a biomarker to predict the efficacy of anti-PD-1/PD-L1 inhibitors in cancer patients. Our meta-analysis explored the relationship between PD-L1 proportion scores, with 1% and 50% cut-off values, and OS and PFS. Analyses revealed a significant response with both 50% and 1% values, but those with scores ≥50% had a lower HR of death than those with values ≥1% (OS, 0.60 vs 0.70; PFS, 0.59 vs 0.74). This means that a much stricter cut-off value (<1%) still fails to demarcate PD-L1 negativity. Additionally, <5%, <10% and <20% values in the OS and PFS analysis was only based on a small number of RCTs, there’s not really much else we can extrapolate from the results. Therefore, these results indicate that PD-L1 expression might be still insufficient to explain different responses with anti-PD-1/PD-L1 inhibitors from more cut-off values.

In addition to PD-L1 expression, *EGFR* and *RAS* mutation have also been identified by previous studies as potential predictors of good anti-PD-1/PD-L1 inhibitor efficacy. Our pooled data revealed that the presence of *EGFR* mutations was not linked with a better OS response to anti-PD/PD-L1 inhibitors, and patients with *RAS* mutations also failed to acquire a survival benefit from anti-PD/PD-L1 inhibitors. Even more surprisingly, the presence of *EGFR* mutations is suggested to be a risk factor for faster progression and recurrence. This might be because EGFR activation leads to the suppression of the anti-tumour immune response via the induction of regulatory T-cells or reduction in the levels of T-cell chemoattractants, facilitating immune system evasion by tumour cells^44^.

As further analysis suggests, improvements in survival with anti-PD-1/PD-L1 inhibitors may show subgroup-level differences. For example, no significantly different OS was observed between males and females, in contrast to studies by Conforti and colleagues^45^ who reported the survival benefit to be sex-dependent. Differently, only male patients showed better PFS. This might be explained by the differences in mutational burden between males and females. As previously described, a higher mutational tumour burden, which has been confirmed to be present in tumours of several histotypes obtained from males, is a stronger predictor of better prognosis^46^. Further, there was an age-related equal OS benefit between those aged above and below 65 years, but without statistical significance in patients aged over 75 years. Ageing is also currently linked to the decreased expression of co-stimulatory signals required for T-cell activation, leading to suppressed immune activation. Our data showed that smokers have an advantage regarding survival benefit from anti-PD-1/PD-L1 inhibitors, which might be due to the low mutational burden and heterogeneity in non-smokers^47^. In a stratified analysis performed on the basis of organ metastasis, we did not observe a negative effect of anti-PD-1/PD-L1 inhibitors in those with liver metastasis for OS, nor a negative influence on PFS in those with CNS metastasis, because the analysis based on organ metastasis was restricted by the number of studies. Additionally, our results demonstrated an increased efficacy of anti-PD-1/PD-L1 inhibitors in patients with multiple types of cancer, but the results were not reliable in patients with other types of cancer, possibly owing to the vast size differences between lung cancer and other cancer types. Our data showed that cancer patients obtained a survival benefit from ICIs, except for targeted PD-L1 (in terms of PFS). Overexpression of PD-1 directly activates multiple immunologic effector cells in a normal physiological environment. PD-L1 is a ligand of PD-1 and is involved in T-cell inhibition, which suppresses cytokine production and T-cell proliferation. In the tumour microenvironment, tumour cells can escape immunological surveillance and T-cell anti-tumour activity by modulating the PD-1/PD-L1 pathway^48^. Therefore, ICIs designed to target the PD-1/PD-L1 pathway could reverse the capacity of cytotoxic T-cells to recognise and attack tumour cells. In the regimen subgroups, a better efficacy of anti-PD-1/PD-L1 inhibitors was observed, both when they were used as a part of combination therapy and single agents. However, the former showed a better survival benefit, with a slightly lower HR for PFS, although the difference was not statistically significant. Results showed that a combination of anti-PD-1/PD-L1 inhibitors and other agents may have a synergetic effect on anti-tumour activity, particularly with respect to the risk of recurrence and progression, which may be relevant for enhancing the sensitivity of other agents relative to PD-L1 negative. Additionally, comparisons with chemotherapy showed that patients failed to respond to anti-PD-1/PD-L1 inhibitors when they were used in combination with targeted therapy, showing that combined therapy with other therapeutic approaches needs to be optimised to improve anti-tumour activity. However, the AEs in combination therapy are still unclear and warrant further investigation. Furthermore, there was no significant difference in the comparison between anti-PD-1/PD-L1 inhibitors and biologics, whether in OS or PFS analysis, it reveals that anti-PD-1/PD-L1 inhibitors failed to provide more survival benefits than biologics, including TKI, anti-angiogenic agents and the like, which indicates anti-PD-1/PD-L1 inhibitors can be compared to a biologics in clinic so far—a an entirely new line of medical research into anti-tumor treatment, but no substitute for biologics.

In the ORR analysis, we found that anti-PD-1/PD-L1 inhibitors had a better ability to control disease in cancer patients. This is mainly because of the different approaches used for tumour shrinkage: in chemotherapy, tumour cells are directly targeted, whereas the effects of anti-PD-1/PD-L1 inhibitors depend on the activation of immune effector cells. Hence, anti-PD-1/PD-L1 inhibitors cannot exert anti-tumour effects until they activate the immune system and consequently generate a series of specific reactions that combat tumour cells; thus, in clinical practice, it takes longer for their effects to be seen^49,50^. However, anti-PD-1/PD-L1 inhibitors have been proven to induce the infiltration of immune effector cells into the tumour, which requires an extended process. Therefore, tumour volumes would initially increase transiently but subsequently shrink during the course of treatment with anti-PD-1/PD-L1 inhibitors, and eventually, the tumour would regress^51,52^. This pseudo progression^53^ weakens the correlation between ORR and anti-PD-1/PD-L1 response.

As suggested by the AE analysis, we found that anti-PD-1/PD-L1 inhibitors caused fewer AEs than conventional therapies. Overall, we observed that patients treated with anti-PD-1/PD-L1 inhibitors had lower risks of all-grade anaemia, neutropenia, diarrhoea, stomatitis, nausea, asthenia, alopecia, neuropathy, fatigue, vomiting, constipation, mucosal inflammation, decreased appetite, decreased neutrophil count, and decreased white-cell count than those in the control group. However, the use of anti-PD-1/PD-L1 inhibitors was associated with a higher incidence of pyrexia, rash, pneumonitis, and pruritus than the use of conventional therapies. The differences in risk were more substantial for all-grade AEs than for high-grade (≥3) AEs, owing to the lower incidence of high-grade AEs in the intervention and control groups. Theoretically, those AEs could be mainly be a result of the nonspecific activation of antigen presenting cells, T-cell infiltration, and reversed immunosuppression^54–56^.

Our study had several limitations. First, the data acquired were based solely on study-level evidence, rather than on results from individual patients, reducing the perceived reliability of the results. Second, we could not exclude the influence of other variables beside treatments that could affect the response to anti-PD-1/PD-L1 inhibitors owing to among-study heterogeneity. Third, we included more studies on lung cancer than on any other type of cancer, and more studies on anti-PD-1 inhibitors than on anti-PD-L1 inhibitors; the subgroup outcomes could be a result of these differences, which in turn makes it impossible to obtain exact conclusions. Furthermore, due to the lack of original data, we were unable to perform subgroup analysis based on more PD-L1 cut-off values (5%, 10% and 20%), other molecular markers (ROS, ALK, or tumour mutation burden), and clinical stages to identify the specific population that stands to benefit from this treatment. Finally, another limitation is that most included studies had an open-label design, and there may thus have been patients in the control group who did not receive drugs in strict accordance with the predefined allocation.

In conclusion, the results of our meta-analysis show that anti-PD-1/PD-L1 inhibitors are more advantageous for the treatment of advanced and metastatic cancer than conventional therapies, especially among male patients, those younger than 65 years of age, current or former smokers, those with no CNS or liver metastasis, those negative for *EGFR* mutations, and those with high PD-L1 expression. Our results also provide reliable evidence of a balance between safety and benefits of anti-PD-1/PD-L1 inhibitors. Further research is required to elucidate the long-term efficacy and safety of anti-PD-1/PD-L1 inhibitors, and also to identify which patients would benefit the most from treatment with these agents.

## Methods

Our meta-analysis was performed in accordance with the PRISMA guidelines^57^ and has been registered in the International Prospective Register of Systematic Reviews (number: CRD42019117020).

### Search strategy and study selection

We performed a literature search for RCTs using PubMed, Embase, and Cochrane databases (Supplementary Method 1). The article search was conducted from the origin of each electronic database to Oct 13, 2019. Two independent investigators retrieved articles searched from the databases and excluded duplicate publications. In addition, we searched for abstracts and presentations from major conferences up to Oct 13, 2019, such as ASCO and ESMO, to make sure that no eligible articles were missed.

To be eligible, studies had to meet the following criteria: (1) must be a prospective phase II or III trial in patients with advanced or metastatic cancer; (2) must provide survival data based on OS and PFS; (3) enrolled patients in the intervention group must have received anti-PD-1 or anti-PD-L1 inhibitors, regardless of dosage and duration; (4) in the intervention group, anti-PD-1 or anti-PD-L1 inhibitors must have been administered alone or in combination with drugs other than anti-CTLA-4 inhibitors; and (5) patients in the control group must have received the control regimen without ICIs. Meanwhile, RCTs were excluded directly if (1) the article was a case report, review, meta-analysis, animal, or *in vitro* study; (2) articles were presented only as meeting abstracts without full text; (3) articles were on studies that were not RCTs; (4) articles were on phase I RCTs; and (5) articles failed to provide data regarding baseline characteristics. Two investigators independently looked through the titles and abstracts of each article to selected the eligible ones in accordance with the criteria mentioned above. They then looked through the full text of the selected articles. All differences of opinions on study selection among investigators were resolved through mutual consultation among all investigators.

### Data extraction and definition

Data extraction was carried out using a predefined format, and all data were also separately extracted by two investigators. Information on study characteristics was extracted from each included study, and included first author, publication date, follow-up, trial design, phase, line of treatment, total number of patients, median age, and drug administration in each group. The primary endpoint was defined as the combination of HRs and 95% CIs for OS and PFS on an ITT basis. The second prespecified endpoint included the RR with 95% CIs for ORRs and AEs. In AEs analysis, we calculated RRs and their 95% CIs based on the number of patients with AEs from each study to assess the therapeutic safety and risks. We also screened supplementary materials from each article to avoid overlooking related data included in appendix.

### Statistical analysis

All final outcomes were calculated using STATA 15 and Review Manager (RevMan) 5.3 by three independent investigators. Results were presented as HRs or RRs with 95% CIs. All statistical tests were two-sided, and a P value less than 0.05 indicated statistical significance. After extracting data from each individual study, STATA 15 was used to pool the time-to-event data and automatically generate forest plots to summarise and visualise HRs with 95% CIs for all included studies. Then, we estimated among-study heterogeneity using the I^2^ test, which enabled us to evaluate the total variability across all studies. We selected a random‐effects model when I^2^ > 50% or *P* < *0*.*05*. Moreover, three fixed knots at 25%, 50%, and 75% from the I² test were applied as predefined indicators of mild, moderate, and high heterogeneity, respectively. Fixed‐effects models were applied to pool data when no obvious heterogeneity was observed. Subgroup and meta-regression analyses were performed based on factors such as sex, age, smoking status, PD-L1 expression, CNS or liver metastasis, and *RAS* or *EGFR* mutation to explore the potential source of heterogeneity among studies and its possible contribution to the main results. Funnel plots were examined for asymmetry to measure the potential publication bias when there were ten or more included studies. Furthermore, Egger’s and Begg’s tests were used to assess publication bias, and *P* < *0*.*05* indicated statistical significance. To estimate the variation in the outcomes owing to different parts of included studies due to methodological bias, sensitivity analysis was conducted by step-wise removal of single studies.

## Supplementary information

Supplementary Material.

## Data Availability

All data generated or analysed during this study are included in this published article and its Supplementary Information files.
