# Supplementary material for: Clinical efficacy and safety of anti-PD-1/PD-L1 inhibitors for the treatment of advanced or metastatic cancer: a systematic review and meta-analysis
Source: Sci Rep. 2020 Feb 7;10:2083. doi: 10.1038/s41598-020-58674-4 (PMC7005709; doi:10.1038/s41598-020-58674-4)

SUPPLEMENTARY MATERIAL

Clinical efficacy and safety of anti-PD-1/PD-L1 inhibitors for the treatment of advanced or metastatic cancer: a systematic review and meta-analysis

Leitao Sun[1, 3*], Leyin Zhang[1*], Jieru Yu[2], Yinan Zhang[1], Xi Pang[1], Chenghao Ma[1], Minhe Shen[3], Shanming Ruan[3#], Harpreet S Wasan[4], Shengliang Qiu[5#]

*These authors contributed equally to this work.
#Corresponding author: Shengliang Qiu, The Fist Affiliated Hospital of Zhejiang Chinese Medical University, Hangzhou 310053, Zhejiang, China. E-mail: shengliang.qiu@zcmu.edu.cn.

Supplementary Method

Supplementary Method 1 - Search strategy for PubMed

Supplementary Method 2 - Risk of Methodological Bias Assessment

Supplementary Table

Supplementary Table 1 - Further Characteristics of the included trial

Supplementary Table 2 - Subgroup analysis of OS

Supplementary Table 3 - Subgroup analysis of PFS

Supplementary Figure

Supplementary Figure 1 - Risk of bias graph

Supplementary Figure 2 - Risk of bias summary

Supplementary Figure 3 - PD-L1 biomarker analysis of OS

Supplementary Figure 4 - PD-L1 biomarker analysis of PFS

Supplementary Figure 5 - ORR analysis of RR and 95%CI based on anti-PD-1/PD-L1 inhibitor used and target spot

Supplementary Figure 6 - ORR analysis of meta-regression analysis based on regimen used and target spot

Supplementary Figure 7 - Sensitivity analysis of OS and PFS

Supplementary Figure 8 - Funnel plot of OS and PFS

Supplementary Figure 9 - Egger's test of OS and PFS

Supplementary Figure 10 - Begg's test of OS and PFS

Supplementary Method 1– Search strategy for PubMed

(((((((((((((((((((((((((Programmed Cell Death 1 Receptor[MeSH Terms]) OR PD-1 Receptor[Title/Abstract]) OR PD1 Receptor[Title/Abstract]) OR Receptor, PD-1[Title/Abstract]) OR Programmed Cell Death 1 Protein[Title/Abstract]) OR Programmed death receptor 1[Title/Abstract]) OR PD-1[Title/Abstract]) OR Programmed death ligand1[Title/Abstract]) OR PD-L1[Title/Abstract]) OR Programmed Cell Death 1 Receptor[Title/Abstract]) OR nivolumab[Title/Abstract]) OR pembrolizumab[Title/Abstract]) OR atezolizumab[Title/Abstract]) OR durvalumab[Title/Abstract]) OR cemiplimab[Title/Abstract]) OR toripalimab[Title/Abstract]) OR sintilimab[Title/Abstract]) OR avelumab[Title/Abstract]) OR Keytruda[Title/Abstract]) OR Opdivo[Title/Abstract]) OR Libtayo[Title/Abstract]) OR Tecentriq[Title/Abstract]) OR Bavencio[Title/Abstract]) OR Imfinzi[Title/Abstract]) OR Camrelizumab[Title/Abstract]) AND ((((((((((((((((((((((((((Neoplasms[MeSH Terms]) OR Neoplasm*[Title/Abstract]) OR tumor*[Title/Abstract]) OR Cancer*[Title/Abstract]) OR malignan*[Title/Abstract]) OR carcinoma*[Title/Abstract]) OR tumour*[Title/Abstract]) OR adenocarcin*[Title/Abstract]) OR Hematologic Neoplasms[MeSH Terms]) OR hematolog* malignan*[Title/Abstract]) OR hematolog* neoplas*[Title/Abstract]) OR lymphom*[Title/Abstract]) OR leukem*[Title/Abstract]) OR hodgkin*[Title/Abstract]) OR lymphogranulomato*[Title/Abstract]) OR histiocy*[Title/Abstract]) OR granulom*[Title/Abstract]) OR non-hodgkin*[Title/Abstract]) OR nonhodgkin*[Title/Abstract]) OR lymphosarcom*[Title/Abstract]) OR reticulosarcom*[Title/Abstract]) OR brill-symmer*[Title/Abstract]) OR plasmacytom*[Title/Abstract]) OR myelom*[Title/Abstract]) OR sezary[Title/Abstract]) OR myelodysplas*[Title/Abstract]) OR aplastic anem*[Title/Abstract]) AND (((((((((((randomized controlled trial[Publication Type]) OR controlled clinical

trial[Publication Type]) OR randomized[Title/Abstract]) OR placebo[Title/Abstract]) OR randomly[Title/Abstract]) OR trial[Title/Abstract]) OR groups[Title/Abstract])) NOT ((animals[MeSH Terms]) NOT ((humans[MeSH Terms]) AND animals[MeSH Terms]))))))

Supplementary Method 2 - Risk of Methodological Bias Assessment

The Cochrane evaluation handbook of RCTs, includes selection bias (random sequence generation and allocation concealment), performance bias (blinding of participants and personnel), detection bias (blinding of outcome assessment), attrition bias (incomplete outcome data), reporting bias (selective reporting) and other bias, was applied to evaluate the quality of all studies. Two independent investigators (CHM and XP) subjectively examined each trial and scored it as high, low, or unclear risk of bias. All disagreements about bias assessment were resolved through discussion and consensus.

Supplementary Table 1- Further Characteristics of the included trial

Author, year	Stage	No. of Male	No. of Female	Median age, years	Follow-up, months	No. of Patients	Median PFS, months	Median OS, months
Horn 2018	Extensive	261 (65%)	142 (35%)	64 (26-90)	13.9 months	201	5.2 (4.4-5.6)	12.3 (10.8-15.9)
						202	4.3 (4.2-4.5)	10.3 (9.3-11.3)
Cohen 2018	II/III/IV	412 (83%)	83 (17%)	60 (54-66)	7.5 months vs 7.1 months	247	2.1 (2.1-2.3)	8.4 (6.4-9.4)
						248	2.3 (2.1-2.8)	6.9 (5.9-8.0)
Schmid 2018	IV	4 (0.4%)	898 (99.6%)	NA (20-86)	12.9 months	451	7.2 (5.6-7.5)	21.3 (17.3-23.4)
						451	5.5 (5.3-5.6)	17.6 (15.9-20.0)
Powles 2018	IV	718 (77%)	213 (23%)	67 (31-88)	17.3 months	467	2.1 (2.1-2.2)	8.6 (7.8-9.6)
						464	4.0 (3.4-4.2)	8.0 (7.2-8.6)
Paz-Ares 2018	IV	455 (81%)	104 (19%)	65 (29-88)	7.8 months	278	6.4 (6.2-8.3)	15.9 (13.2-NA)
						281	4.8 (4.3-5.7)	11.3 (9.5-14.8)
Larkin 2018	IIIC/IV	261 (64%)	144 (36%)	NA (23-88)	approximately 2 years	272	3.1 (2.3-3.5)	15.7 (12.9-19.9)
						133	3.7 (2.3-5.3)	14.4 (11.7-18.2)
Herbst 2016	IV	634 (61%)	399 (39%)	NA (56-69)	13.1 months	344	3.9 (3.1-4.1)	10.4 (9.4-11.9)
						346	4.0 (2.7-4.3)	12.7 (10.0-17.3)
						343	4.0 (3.1-4.2)	8.5 (7.5-9.8)
Gandhi 2018	IV	363 (59%)	253 (41%)	NA (34-84)	10.5 months	410	8.8 (7.6-9.2)	NA
						206	4.9 (4.7-5.5)	11.3 (8.7-15.1)
Fehrenbacher 2018	IV	758 (62%)	467 (38%)	NA (25-85)	26months	613	2.7 (2.4-2.9)	13.3 (11.3-14.9)
						612	3.8 (3.3-4.1)	9.5 (8.6-10.9)
Barlesi 2018	IIIB/ IV	542 (68%)	250 (32%)	NA (57-69)	18.3 months	396	2.8 (2.7-3.5)	10.5 (9.2-12.9)
						396	4.2 (3.3–5.2)	9.9 (8.1-11.8)
Antonia 2018	III	500 (70%)	213 (30%)	64 (23-90)	25.2 months	476	17.2 (13.1-23.9)	NA (34.7–NA)
						237	5.6 (4.6-7.7)	28.7 (22.9–NA)
Reck 2016	IV	187 (61%)	118 (39%)	NA (33-90)	11.2 months	154	10.3 (6.7-NA)	NA
						151	6.0 (4.2-6.2)	NA
Kang 2017	IV	348 (71%)	145 (29%)	NA (53-69)	8.87 months vs 8.59 months	330	1.61 (1.54-2.30)	5.26 (4.60-6.37)
						163	1.45 (1.45-1.54)	4.14 (3.42-4.86)
Carbone 2017	IV	332 (61%)	209 (39%)	64 (29-89)	13.5months	271	4.2 (3.1-5.5)	13.7 (11.8-15.4)
						270	5.8 (5.4-6.9)	13.8 (11.0-17.0)
Bellmunt 2017	IV	402 (74%)	140 (26%)	NA (26-88)	14.1 months	270	2.1 (2.0-2.2)	10.3 (8.0-11.8)
						272	3.3 (2.3-3.5)	7.4 (6.1-8.3)
Borghaei 2015	IIIB/IV	319 (55%)	263 (45%)	62 (21-85)	17.2 months+	292	2.3 (2.2–3.3)	12.2 (9.7–15.0)
						290	4.2 (3.5–4.9)	9.4 (8.1–10.7)
Brahmer 2015	IIIB/IV	208 (76%)	64 (24%)	63 (39-85)	11 months +	135	3.5 (2.1-4.9)	9.2 (7.3-13.3)
						137	2.8 (2.1-3.5)	6.0 (5.1-7.3)
Motzer 2015	IV	619 (75%)	202 (25%)	62 (18-88)	14 months +	410	4.6 (3.7-5.4)	25.0 (21.8-NA)
						411	4.4 (3.7-5.5)	19.6 (17.6-23.1)
Fehrenbacher 2016	IV	169 (59%)	118 (41%)	62 (36-84)	13 months +	144	2.7 (2.0-4.1)	12.6 (9.7-16.4)
						143	3.0 (2.8-4.1)	9.7 (8.6-12.0)
Ferris 2016	IV	300 (83%)	61 (17%)	60 (28-83)	5.1 months	240	2.0 (1.9-2.1)	7.5 (5.5-9.1)
						121	2.3 (1.9-3.1)	5.1 (4.0-6.0)
Socinski 2018	IV	479 (60%)	321 (40%)	63 (31-90)	9.5 momths+	400	8.3 (7.9–9.8)	NA
						400	6.8 (6.0–7.1)	NA
Ascierto 2018	III/IV	246 (59%)	172 (41%)	NA (18-87)	38.4 momths+ vs 38.5 months+	210	5.1 (3.5–12.2)	37.5 (25.5-NA)
						208	2.2 (2.1–2.5)	11.2 (9.6-13.0)
Bang 2018	IV	267 (72%)	104 (28%)	NA (18-86)	10.6 months	185	4.6 (3.6–5.7)	NA
						186	5.0 (4.5–6.3)	NA
Mateos 2019	III	155 (62%)	94 (38%)	NA (60-74)	8.1 months	125	5.6 (3.7–7.5)	NA (12.9-NA)
						124	8.4 (5.9–NA)	15.2 (12.7-NA)
Usmani 2019	III	151 (50%)	150(50%)	74 (70-79)	6.6 months	151	NA	NA
						150	NA	NA
Borghaei 2019	IIIB/IV	NA	NA	NA	23.9 months	60	24.0 (8.5-NA)	NA (24.5-NA)
						63	9.3 (6.2-14.9)	21.1 (14.9-NA)
Eng 2019	IV	217 (60%)	146 (40%)	NA (51-67)	7.3 months	183	1.91 (1.87–1.97)	8.87 (7.00-10.61)
						90	1.94 (1.91–2.10)	7.10 (6.05-10.05)
						90	2.00 (1.87–3.61)	8.51 (6.41–10.71)
Fradet 2019	IV	402 (74%)	140 (26%)	NA (26-88)	27.7 months	266	2.1 (2.0–2.2)	10.1 (8.0–12.3)
						255	3.3 (2.4–3.6)	7.3 (6.1–8.1)
Hamid 2017	IV	327 (61%)	213 (39%)	NA (15-89)	28 months	180	NA	13.4 (11.0-16.4)
						181	NA	14.7 (11.3-19.5)
						179	NA	11.0 (8.9-13.8)
Mok 2019	IV	902 (71%)	372 (29%)	63 (59-69)	12.8 months	637	7.1 (5.9-9.0)	NA
						637	6.4 (96.1-6.9)	NA
Motzer 2019	IV	660 (74%)	226 (25%)	NA	10.8 momths vs 8.6 months	442	13.8 (11.1-NA)	NA
						424	8.4 (6.9-11.1)	NA
Weber 2015	IV	261 (64%)	144 (36%)	NA (23-88)	8.4 months	272	4.7 (2.3-6.5)	NA
						133	4.2 (2.1-6.3)	NA
West 2019	IV	415 (57%)	308 (43%)	NA (18-86)	18.5 momths vs 19.2 months	483	7.0 (6.3–7.3)	18.1 (15.3–20.8)
						240	5.6 (4.5–5.9)	13.9 (12.0–18.2)
Brian 2019	IV	669 (73%)	246 (27%)	NA (54069)	15 months	454	11.2 (9.6–13.3)	33.6 (29.0–NA)
						461	8.4 (7.5-9.7)	34.9 (27.8–NA)
Rini 2019	IV	628(73%)	233 (27%)	NA (26-90)	12.8 months	432	15.1 (12.6-24.9)	NA
						429	11.1 (8.7-12.5)	NA

Abbreviations: †Represents minimum follow-up time between the start of the study (the first visit of the first enrolled patient) and the end of the study (the last visit of the last enrolled patient).

Supplementary Table 2- Subgroup analysis: Subgroup analysis of pooled hazard ratios and 95%CI of OS for patients with advanced or metastatic cancer assigned to intervention treatment, compared with those assigned to control treatment.

Analysis	N	Random-effects model		Fixed-effects model		Heterogeneity	
		HR (95% CI)	*P*	HR (95% CI)	*P*	I^2	*Ph*
**Histotype**	34	0.76 (0.71, 0.82)	≤0.001	0.77 (0.74, 0.80)	≤0.001	68.2%	≤0.001
HNC	2	0.77 (0.65, 0.91)	0.003	0.77 (0.65, 0.91)	0.003	0.0%	0.488
GC/GEJC	2	0.83 (0.48, 1.44)	0.509	0.82 (0.71, 0.96)	0.013	92.1%	≤0.001
UC	3	0.77 (0.68, 0.87)	≤0.001	0.78 (0.70, 0.86)	≤0.001	26.0%	0.256
NSCLC	15	0.72 (0.66, 0.80)	≤0.001	0.75 (0.71, 0.80)	≤0.001	63.1%	≤0.001
MM	2	1.75 (1.10, 2.78)	0.018	1.75 (1.10, 2.78)	0.018	0.0%	0.621
Mm	4	0.69 (0.50, 0.96)	0.029	0.68 (0.59, 0.78)	≤0.001	82.0%	≤0.001
RCC	4	0.74 (0.59, 0.93)	0.010	0.78 (0.68, 0.88)	≤0.001	64.0%	0.040
CRC	2	1.08 (0.85, 1.37)	0.533	1.08 (0.85, 1.37)	0.533	0.0%	0.479
**Regimen**	36	0.76 (0.71, 0.82)	≤0.001	0.77 (0.74, 0.80)	≤0.001	66.7%	≤0.001
combination therapy	12	0.78 (0.66, 0.91)	0.002	0.77 (0.71, 0.83)	≤0.001	71.8%	≤0.001
Monotherapy	24	0.76 (0.70, 0.82)	≤0.001	0.77 (0.74, 0.81)	≤0.001	65.2%	≤0.001
**Combination Drug**	12	0.78 (0.66, 0.91)	0.002	0.77 (0.71, 0.83)	≤0.001	71.8%	≤0.001
Chemotherapy	6	0.68 (0.57, 0.81)	≤0.001	0.70 (0.64, 0.78)	≤0.001	61.9%	0.022
Targeted therapy	6	0.94 (0.71, 1.26)	0.694	0.88 (0.77, 1.01)	0.066	73.7%	0.002
**Treatment in control group**	34	0.76 (0.71, 0.82)	≤0.001	0.77 (0.74, 0.80)	≤0.001	68.5%	≤0.001
Chemotherapy	24	0.74 (0.68, 0.80)	≤0.001	0.76 (0.73, 0.80)	≤0.001	68.0%	≤0.001
Placebo	2	0.64 (0.53, 0.77)	≤0.001	0.64 (0.53, 0.77)	≤0.001	0.0%	0.729
Biologics	8	0.93 (0.74, 1.16)	0.496	0.87 (0.78, 0.98)	0.017	70.7%	≤0.001
**Age**	20	0.76 (0.70, 0.82)	≤0.001	0.77 (0.73, 0.81)	≤0.001	55.4%	≤0.001
<65yr	20	0.77 (0.69, 0.87)	≤0.001	0.79 (0.74, 0.84)	≤0.001	67.6%	≤0.001
≥65yr	15	0.76 (0.67, 0.84)	≤0.001	0.76 (0.70, 0.82)	≤0.001	38.1%	0.067
≥65 to <75yr	5	0.64 (0.54, 0.77)	≤0.001	0.64 (0.54, 0.77)	≤0.001	0.0%	0.615
≥75yr	6	0.88 (0.53, 1.46)	0.616	0.91 (0.65, 1.26)	0.553	57.1%	0.040
**Sex**	19	0.74 (0.69, 0.80)	≤0.001	0.75 (0.71, 0.79)	≤0.001	52.7%	≤0.001
Male	19	0.74 (0.69, 0.79)	≤0.001	0.74 (0.70, 0.79)	≤0.001	19.9%	0.216
Female	19	0.75 (0.63, 0.88)	≤0.001	0.77 (0.70, 0.84)	≤0.001	67.5%	≤0.001
**ECOG**	18	0.77 (0.71, 0.83)	≤0.001	0.77 (0.73, 0.81)	≤0.001	47.5%	≤0.001
0	18	0.81 (0.71, 0.93)	0.002	0.81 (0.74, 0.89)	≤0.001	52.0%	0.005
1	16	0.74 (0.68, 0.80)	≤0.001	0.75 (0.70, 0.80)	≤0.001	40.5%	0.047
**Smoking**	16	0.77 (0.70, 0.84)	≤0.001	0.78 (0.73, 0.82)	≤0.001	51.6%	≤0.001
Current/Former	16	0.75 (0.68, 0.83)	≤0.001	0.76 (0.71, 0.81)	0.020	49.6%	0.013
Never	10	0.81 (0.66, 1.00)	0.045	0.85 (0.75, 0.98)	≤0.001	53.6%	0.022
**Line**	36	0.76 (0.71, 0.82)	≤0.001	0.77 (0.74, 0.80)	≤0.001	66.7%	≤0.001
First-line	14	0.72 (0.62, 0.84)	≤0.001	0.75 (0.70, 0.80)	≤0.001	77.5%	≤0.001
Subsequent line	22	0.78 (0.73, 0.85)	≤0.001	0.78 (0.74, 0.82)	≤0.001	54.8%	≤0.001
**Masking**	36	0.76 (0.71, 0.82)	≤0.001	0.77 (0.74, 0.80)	≤0.001	66.7%	≤0.001
Double-blind	9	0.64 (0.56, 0.74)	≤0.001	0.65 (0.60, 0.71)	≤0.001	61.3%	0.008
Open-label	27	0.81 (0.75, 0.87)	≤0.001	0.81 (0.77, 0.84)	≤0.001	60.0%	≤0.001
**Target spot**	36	0.76 (0.71, 0.82)	≤0.001	0.77 (0.74, 0.80)	≤0.001	66.7%	≤0.001
PD-L1	13	0.86 (0.80, 0.92)	≤0.001	0.86 (0.81, 0.91)	≤0.001	26.8%	0.174
PD-1	23	0.71 (0.64, 0.78)	≤0.001	0.71 (0.68, 0.75)	≤0.001	68.1%	≤0.001
**Anti-PD-1/PD-L1 inhibitor**	35	0.76 (0.71, 0.82)	≤0.001	0.77 (0.74, 0.80)	≤0.001	67.5%	≤0.001
Atezolizumab	9	0.84 (0.78, 0.91)	≤0.001	0.84 (0.78, 0.90)	≤0.001	11.6%	0.338
Pembrolizumab	15	0.70 (0.63, 0.79)	≤0.001	0.71 (0.67, 0.76)	≤0.001	60.2%	≤0.001
Nivolumab	8	0.71 (0.59, 0.86)	≤0.001	0.72 (0.66, 0.79)	≤0.001	79.3%	≤0.001
Avelumab	3	0.94 (0.79, 1.12)	0.484	0.94 (0.83, 1.08)	0.387	40.0%	0.189
**CNS Metastasis**	6	0.71 (0.61, 0.82)	≤0.001	0.73 (0.67, 0.79)	≤0.001	56.5%	0.011
Yes	5	0.78 (0.48, 1.25)	0.303	0.73 (0.57, 0.94)	0.016	70.0%	0.010
No	6	0.70 (0.61, 0.80)	≤0.001	0.73 (0.67, 0.80)	≤0.001	48.3%	0.085
**Liver Metastasis**	4	0.75 (0.68, 0.83)	≤0.001	0.75 (0.68, 0.83)	≤0.001	0.0%	0.564
Yes	4	0.81 (0.68, 0.96)	0.017	0.81 (0.68, 0.96)	0.017	0.0%	0.854
No	4	0.71 (0.62, 0.82)	≤0.001	0.72 (0.64, 0.82)	≤0.001	22.1%	0.278
**RAS**	4	0.95 (0.81, 1.11)	0.498	0.95 (0.81, 1.11)	0.498	0.0%	0.547
Mutant	4	0.87 (0.63, 1.21)	0.408	0.89 (0.69, 1.14)	0.346	40.0%	0.172
Wildtype	4	0.99 (0.81, 1.20)	0.900	0.99 (0.81, 1.20)	0.900	0.0%	0.920
**EGFR**	4	0.74 (0.65, 0.86)	≤0.001	0.73 (0.66, 0.80)	≤0.001	38.6%	0.135
Mutant	3	1.11 (0.80, 1.52)	0.538	1.11 (0.80, 1.52)	0.538	0.0%	0.744
Wildtype	4	0.70 (0.63, 0.77)	≤0.001	0.70 (0.63, 0.77)	≤0.001	0.0%	0.575

Abbreviations: NSCLC, Non-Small Cell Lung Cancer; SCLC, Small Cell Lung Cancer; Mm, Melanoma; RCC, Renal-Cell Carcinoma; UC, Urothelial Carcinoma; GC/GEJC, Gastric or Gastro-oesophageal Junction Cancer; BC, Breast Cancer; HNC, Head-and-Neck Cancer, MM, Multiple Myeloma.

Supplementary Table 3- Subgroup analysis: Subgroup analysis of pooled hazard ratios and 95%CI of PFS for patients with advanced or metastatic cancer assigned to intervention treatment, compared with those assigned to control treatment.

Analysis	N	Random-effects model		Fixed-effects model		Heterogeneity	
		HR (95% CI)	*P*	HR (95% CI)	*P*	I^2	*Ph*
**Histotype**	35	0.81 (0.73, 0.90)	≤0.001	0.82 (0.79, 0.85)	≤0.001	88.6%	≤0.001
HNC	2	0.93 (0.80, 1.08)	0.357	0.93 (0.80, 1.08)	0.357	0.0%	0.629
GC/GEJC	2	1.02 (0.36, 2.87)	0.973	0.99 (0.85, 1.15)	0.869	97.8%	≤0.001
UC	2	0.97 (0.85, 1.11)	0.660	0.97 (0.85, 1.11)	0.660	0.0%	0.882
NSCLC	16	0.75 (0.65, 0.87)	≤0.001	0.81 (0.77, 0.85)	≤0.001	89.3%	≤0.001
MM	2	1.44 (1.05, 1.97)	0.026	1.44 (1.05, 1.97)	0.026	0.0%	0.127
Mm	5	0.60 (0.43, 0.83)	0.002	0.56 (0.49, 0.63)	≤0.001	83.0%	≤0.001
RCC	4	0.78 (0.69, 0.88)	≤0.001	0.79 (0.72, 0.86)	≤0.001	47.3%	0.127
CRC	2	1.31 (1.05, 1.62)	0.014	1.31 (1.05, 1.62)	0.014	0.0%	0.632
**Regimen**	37	0.81 (0.73, 0.89)	≤0.001	0.82 (0.79, 0.85)	≤0.001	88.0%	≤0.001
Combination therapy	13	0.75 (0.65, 0.86)	≤0.001	0.72 (0.68, 0.76)	≤0.001	80.4%	≤0.001
Monotherapy	24	0.84 (0.74, 0.95)	0.007	0.87 (0.84, 0.91)	≤0.001	89.0%	≤0.001
**Combination Drug**	12	0.76 (0.66, 0.89)	≤0.001	0.74 (0.69, 0.78)	≤0.001	80.6%	≤0.001
Chemotherapy	6	0.64 (0.55, 0.76)	≤0.001	0.67 (0.62, 0.72)	≤0.001	70.9%	0.004
Targeted therapy	6	0.94 (0.74, 1.19)	0.602	0.84 (0.76, 0.92)	≤0.001	81.2%	≤0.001
**Treatment in control group**	34	0.81 (0.73, 0.90)	≤0.001	0.82 (0.80, 0.85)	≤0.001	88.4%	≤0.001
Chemotherapy	24	0.79 (0.69, 0.90)	≤0.001	0.84 (0.80, 0.87)	≤0.001	89.5%	≤0.001
Placebo	2	0.55 (0.47, 0.65)	≤0.001	0.55 (0.48, 0.64)	≤0.001	9.9%	0.292
Biologics	8	0.97 (0.80, 1.17)	0.730	0.87 (0.81, 0.95)	≤0.001	79.9%	≤0.001
**Age**	163	0.83 (0.73, 0.95)	0.006	0.82 (0.77, 0.87)	≤0.001	78.0%	≤0.001
<65yr	13	0.82 (0.67, 1.02)	0.078	0.81 (0.75, 0.88)	≤0.001	86.3%	≤0.001
≥65yr	11	0.84 (0.69, 1.01)	0.067	0.83 (0.75, 0.92)	≤0.001	69.9%	≤0.001
≥65 to <75yr	2	0.71 (0.39, 1.29)	0.264	0.78 (0.61, 1.01)	0.063	78.1%	0.033
≥75yr	3	0.98 (0.52, 1.85)	0.956	0.99 (0.63, 1.56)	0.971	46.4%	0.155
**Sex**	11	0.78 (0.67, 0.92)	0.002	0.79 (0.74, 0.84)	≤0.001	83.1%	≤0.001
Male	11	0.75 (0.63, 0.90)	0.002	0.76 (0.71, 0.82)	≤0.001	80.2%	≤0.001
Female	11	0.82 (0.61, 1.11)	0.196	0.83 (0.75, 0.93)	≤0.001	86.2%	≤0.001
**ECOG**	12	0.84 (0.71, 0.99)	0.036	0.81 (0.76, 0.86)	≤0.001	83.7%	≤0.001
0	12	0.91 (0.67, 1.22)	0.530	0.90 (0.81, 1.00)	0.047	86.7%	≤0.001
1	11	0.77 (0.64, 0.93)	0.005	0.76 (0.70, 0.83)	≤0.001	78.7%	≤0.001
**Smoking**	8	0.79 (0.63, 0.97)	0.027	0.75 (0.68, 0.82)	≤0.001	78.5%	≤0.001
Current/Former	8	0.72 (0.57, 0.89)	0.003	0.72 (0.65, 0.79)	≤0.001	77.4%	≤0.001
Never	5	1.00 (0.55, 1.79)	0.991	0.90 (0.73, 1.12)	0.352	81.2%	≤0.001
**Line**	37	0.81 (0.73, 0.89)	≤0.001	0.82 (0.79, 0.85)	≤0.001	88.0%	≤0.001
First-line	15	0.70 (0.60, 0.81)	≤0.001	0.74 (0.70, 0.78)	≤0.001	88.0%	≤0.001
Subsequent line	22	0.89 (0.79, 1.01)	0.081	0.89 (0.85, 0.93)	≤0.001	86.4%	≤0.001
**Masking**	37	0.81 (0.73, 0.89)	≤0.001	0.82 (0.79, 0.85)	≤0.001	88.0%	≤0.001
Double-blind	9	0.57 (0.49, 0.67)	≤0.001	0.60 (0.56, 0.64)	≤0.001	78.7%	≤0.001
Open-label	28	0.91 (0.82, 1.00)	0.054	0.90 (0.87, 0.94)	≤0.001	82.7%	≤0.001
**Target spot**	37	0.81 (0.73, 0.89)	≤0.001	0.82 (0.79, 0.85)	≤0.001	88.0%	≤0.001
PD-L1	13	0.88 (0.75, 1.04)	0.143	0.85 (0.81, 0.89)	≤0.001	90.1%	≤0.001
PD-1	24	0.77 (0.68, 0.87)	≤0.001	0.80 (0.76, 0.83)	≤0.001	86.8%	≤0.001
**Anti-PD-1/PD-L1 inhibitor**	36	0.82 (0.74, 0.90)	≤0.001	0.83 (0.80, 0.86)	≤0.001	87.5%	≤0.001
Atezolizumab	9	0.86 (0.74, 1.00)	0.045	0.83 (0.78, 0.88)	≤0.001	82.5%	≤0.001
Pembrolizumab	15	0.76 (0.65, 0.89)	≤0.001	0.80 (0.75, 0.84)	≤0.001	87.8%	≤0.001
Nivolumab	9	0.78 (0.63, 0.98)	0.029	0.80 (0.75, 0.87)	≤0.001	86.7%	≤0.001
Avelumab	3	1.11 (0.68, 1.83)	0.675	1.09 (0.97, 1.22)	0.163	94.4%	≤0.001
**CNS Metastasis**	5	0.65 (0.54, 0.80)	≤0.001	0.68 (0.62, 0.75)	≤0.001	66.9%	0.002
Yes	4	0.64 (0.42, 0.97)	0.036	0.62 (0.46, 0.84)	0.002	41.0%	0.166
No	5	0.66 (0.52, 0.83)	≤0.001	0.69 (0.62, 0.77)	≤0.001	78.6%	≤0.001
**RAS**	3	1.27 (1.06, 1.51)	0.008	1.27 (1.06, 1.51)	0.008	0.0%	0.423
Mutant	3	1.09 (0.82, 1.43)	0.563	1.09 (0.84, 1.41)	0.532	11.0%	0.325
Wildtype	3	1.45 (1.14, 1.84)	0.002	1.45 (1.14, 1.84)	0.002	0.0%	0.929
**EGFR**	2	1.02 (0.77, 1.36)	0.881	0.89 (0.78, 1.01)	0.070	68.6%	0.023
Mutant	2	1.57 (1.07, 2.31)	0.022	1.57 (1.07, 2.31)	0.022	0.0%	0.621
Wildtype	2	0.83 (0.73, 0.95)	0.007	0.83 (0.73, 0.95)	0.007	0.0%	1.000

Abbreviations: NSCLC, Non-Small Cell Lung Cancer; SCLC, Small Cell Lung Cancer; Mm, Melanoma; RCC, Renal-Cell Carcinoma; UC, Urothelial Carcinoma; GC/GEJC, Gastric or Gastro-oesophageal Junction Cancer; BC, Breast Cancer; HNC, Head-and-Neck Cancer, MM, Multiple Myeloma.

Supplementary Figure 1- Risk of bias graph: Review authors' judgments about each risk of bias item presented as percentages across all included studies.

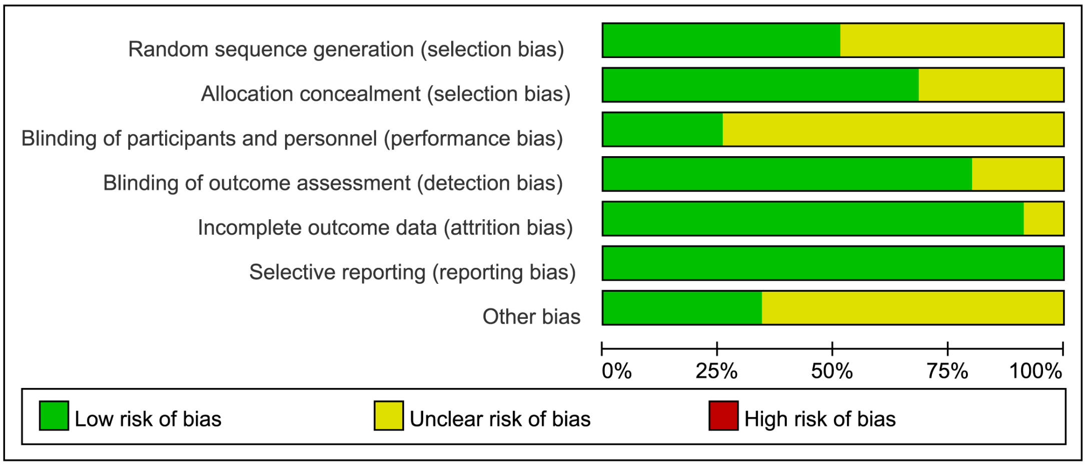

Supplementary Figure 2- Risk of bias summary: Review authors' judgments about each risk of bias item for each included study.

	Random sequence generation (selection bias)	Allocation concealment (selection bias)	Blinding of participants and personnel (performance bias)	Blinding of outcome assessment (detection bias)	Incomplete outcome data (attrition bias)	Selective reporting (reporting bias)	Other bias
Antonia 2018	?	+	+	+	+	+	?
Ascierto2018	?	+	+	?	+	+	?
Bang 2018	+	?	?	+	+	+	?
Barlesi 2018	+	+	?	+	+	+	+
Bellmunt 2017	?	+	?	+	+	+	+
Borghaei 2015	?	?	?	+	+	+	?
Borghaei 2019	?	?	?	+	+	+	?
Brahmer 2015	?	?	?	+	+	+	+
Brian 2019	+	+	?	+	+	+	?
Carbone 2017	?	?	?	+	+	+	?
Cohen 2018	+	+	?	?	+	+	+
Eng 2019	+	+	?	?	+	+	?
Fehrenbacher 2016	+	+	?	+	+	+	+
Fehrenbacher 2018	?	+	?	?	+	+	+
Ferris 2016	?	+	?	+	+	+	+
Fradet 2019	?	?	?	?	+	+	?
Gandhi 2018	+	+	+	+	+	+	+
Hamid 2017	?	+	+	?	+	+	?
Herbst 2016	+	+	?	+	+	+	+
Horn 2018	+	+	+	+	?	+	+
Kang 2017	+	+	+	+	+	+	?
Larkin 2018	?	?	?	+	+	+	?
Mateos 2019	+	+	?	+	+	+	?
Mok 2019	+	+	?	?	+	+	?
Motzer 2015	?	?	?	+	+	+	?
Motzer 2019	?	?	?	+	+	+	?
Paz-Ares 2018	+	+	+	+	?	+	?
Powles 2018	+	+	+	+	+	+	?
Reck 2016	?	+	?	+	+	+	+
Rini 2019	?	?	?	+	+	+	?
Schmid 2018	+	+	+	+	+	+	?
Socinski 2018	?	?	?	+	+	+	+
Usmani 2019	+	+	?	+	+	+	?
Weber 2015	+	+	?	+	+	+	?
West 2019	+	+	?	+	?	+	?

Supplementary Figure 3- PD-L1 biomarker analysis: Analysis of PD-L1 expression pooled hazard ratios and 95%CI of OS with cut-off of 1% (A) and 50% (B) for patients with advanced or metastatic cancer assigned to intervention treatment, compared with those assigned to control treatment.

A

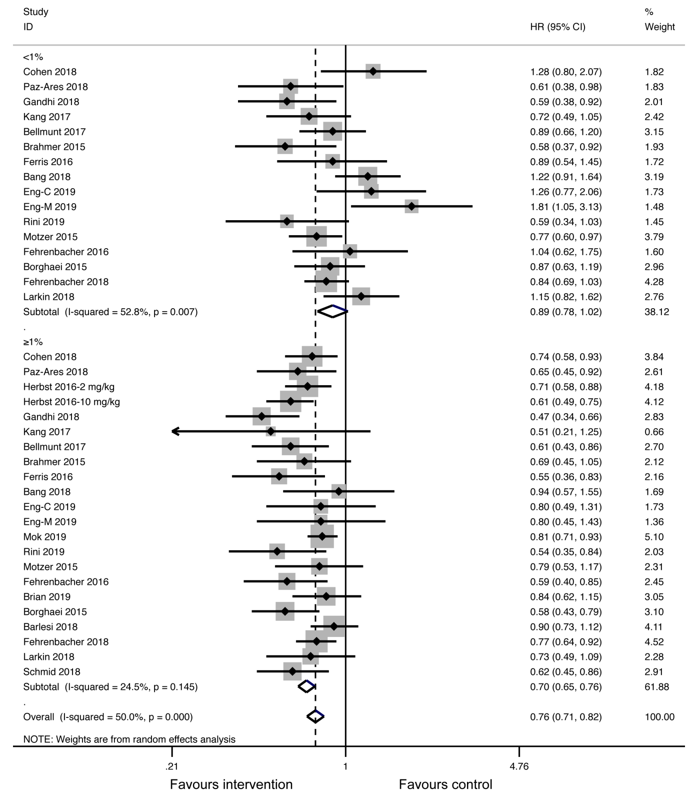

B

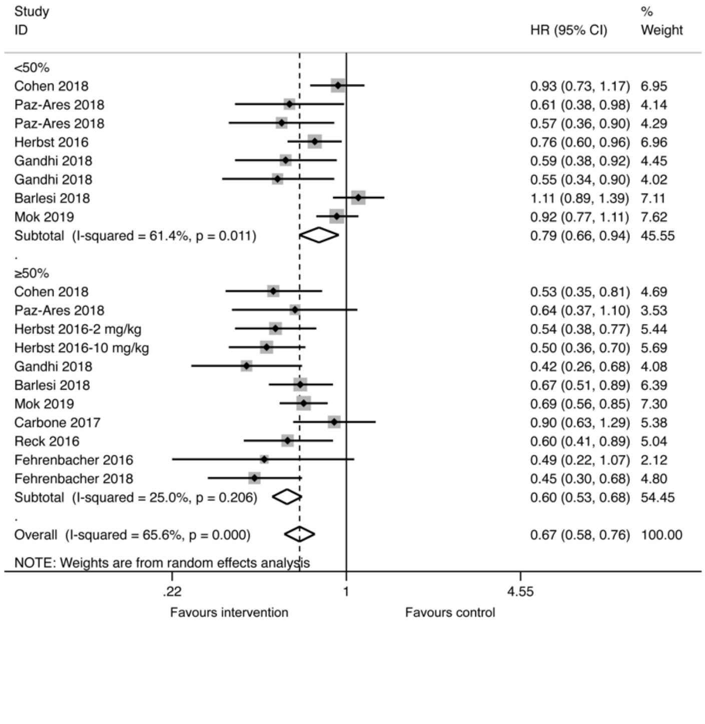

Supplementary Figure 4- PD-L1 biomarker analysis: Analysis of PD-L1 expression pooled hazard ratios and 95%CI of PFS with cut-off of 1% (A) and 50% (B) for patients with advanced or metastatic cancer assigned to intervention treatment, compared with those assigned to control treatment.

A

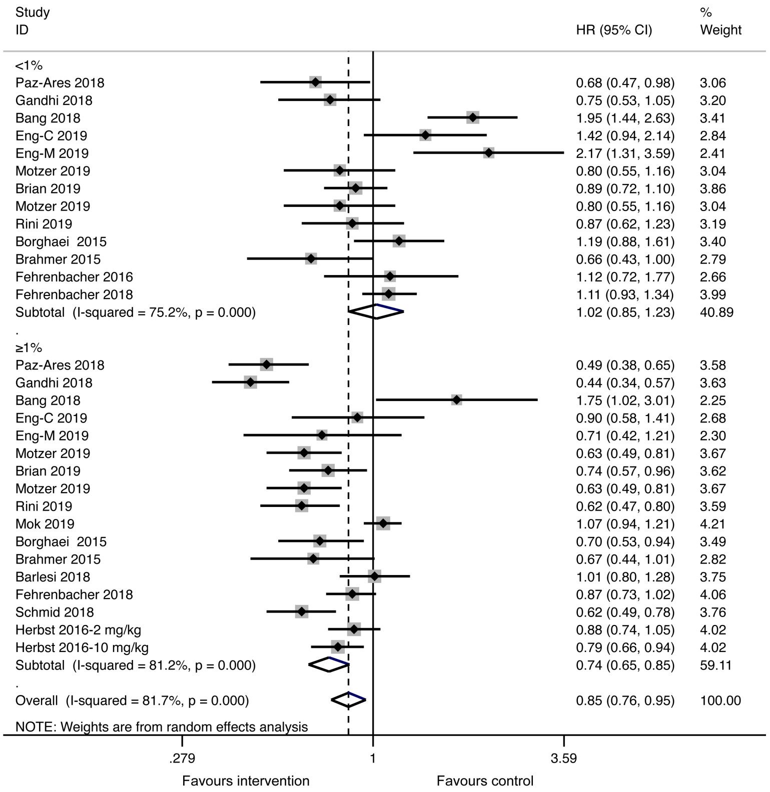

B

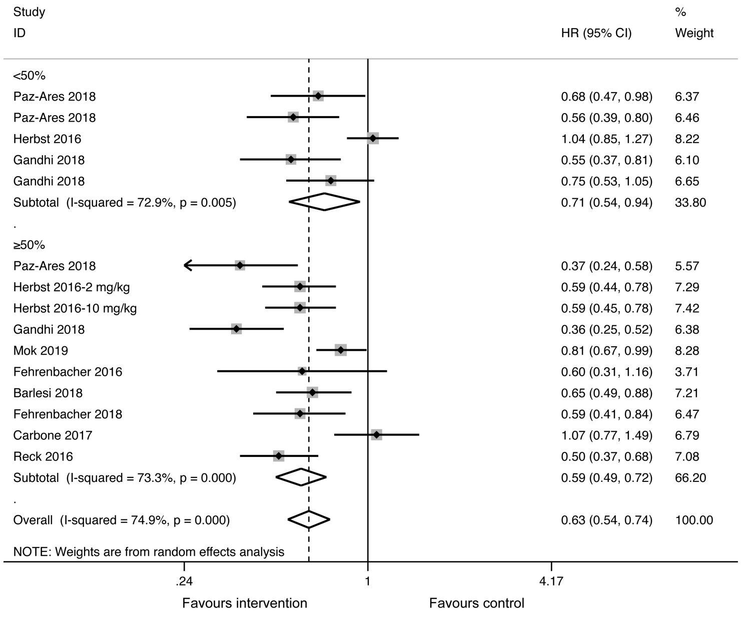

Supplementary Figure 5- ORR analysis: Analysis of ORR pooled relative ratios and 95%CI for patients with advanced or metastatic cancer who received anti-PD-1/PD-L1 inhibitors compared to control therapies based on anti-PD-1/PD- L1 inhibitor used (A) and target spot (B).

A

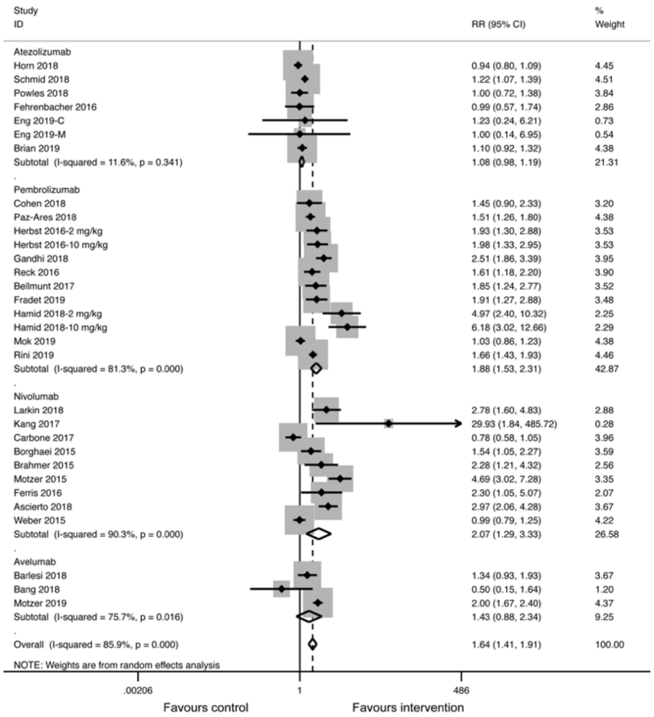

B

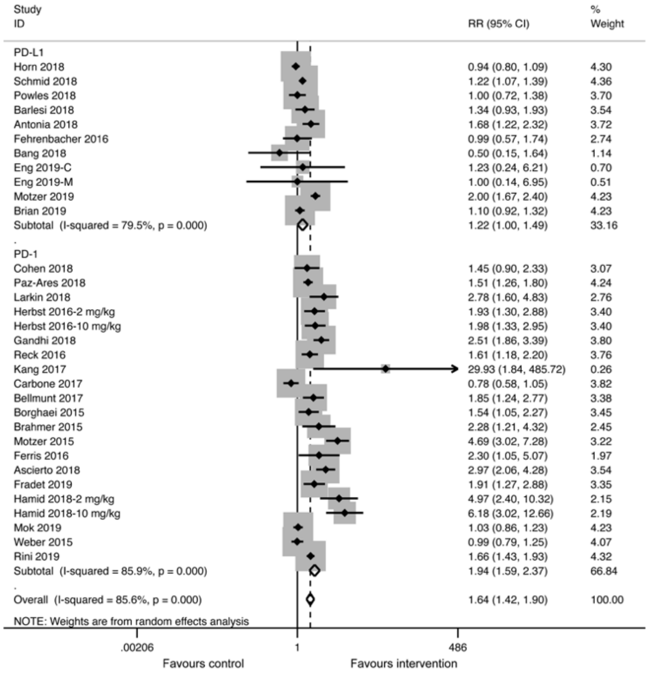

Supplementary Figure 6- ORR analysis: Meta-regression analysis of ORR for patients with advanced or metastatic cancer who received anti-PD-1/PD-L1 inhibitors compared to control therapies based on regimen used (A) and target spot (B).

A

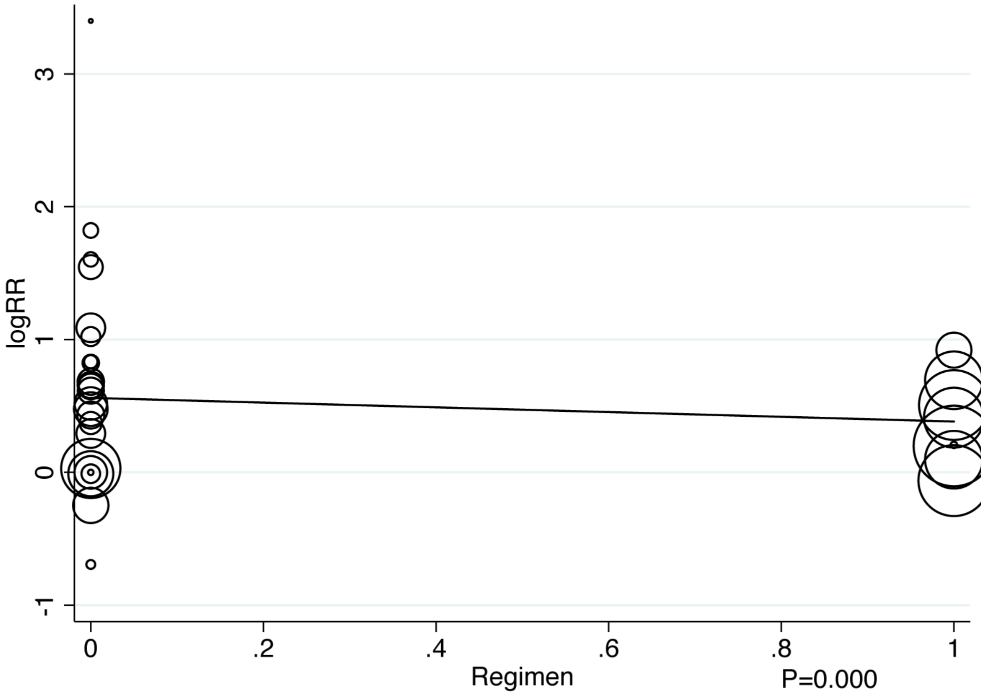

B

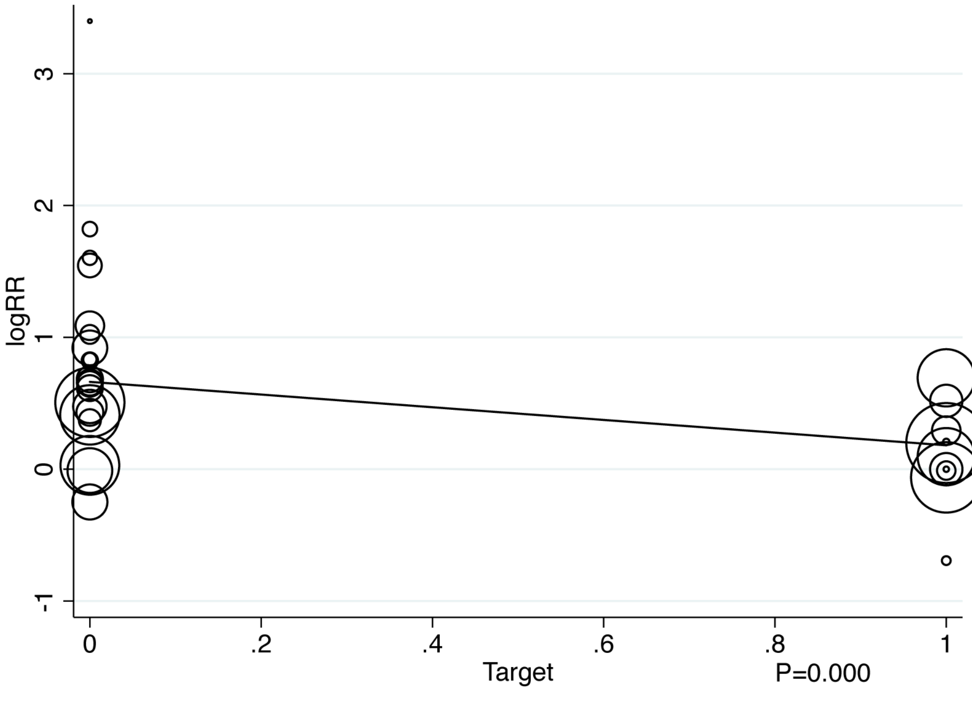

Supplementary Figure 7- Sensitivity analysis: Sensitivity analysis of OS (A) and PFS (B) in included RCTs for the robustness of findings to different aspects of the trials methodology.

A

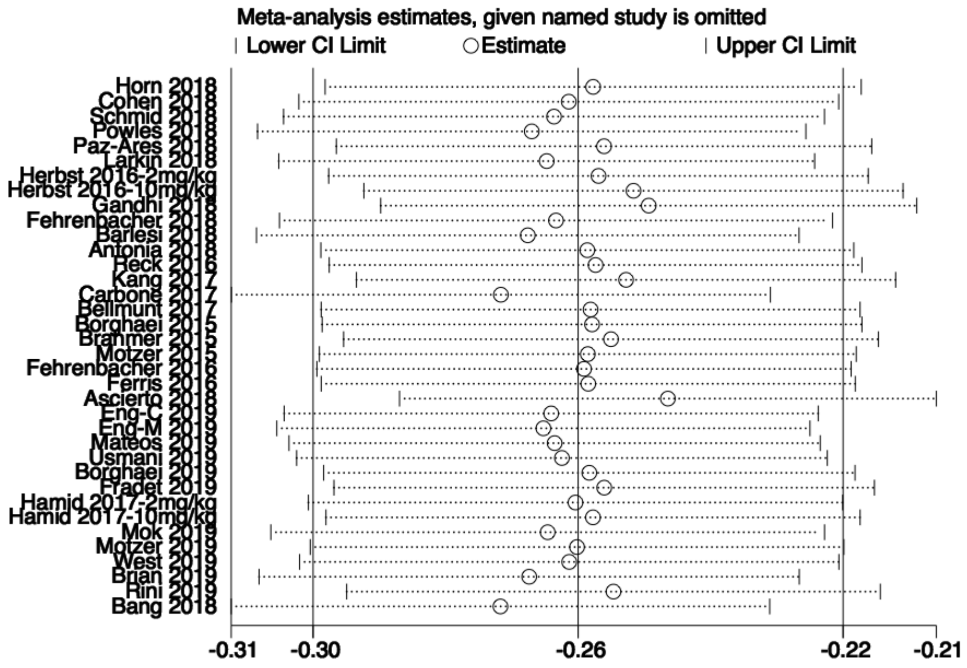

B

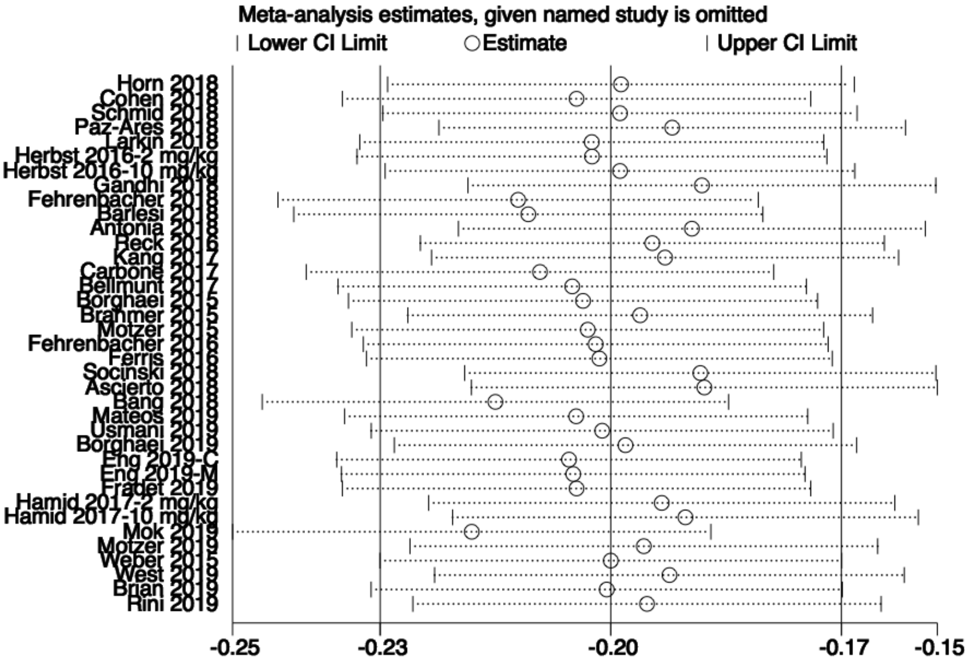

Supplementary Figure 8- Funnel plot: Funnel plot of OS (A) and PFS (B) from in included RCTs for the visual detection of systematic publication bias and small study effect.

A

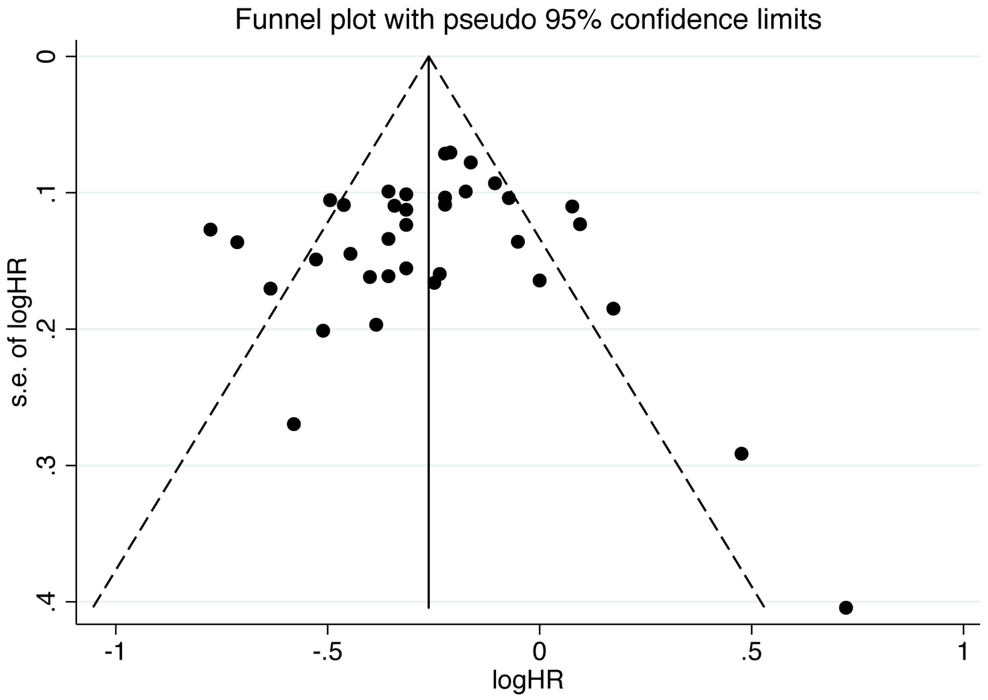

B

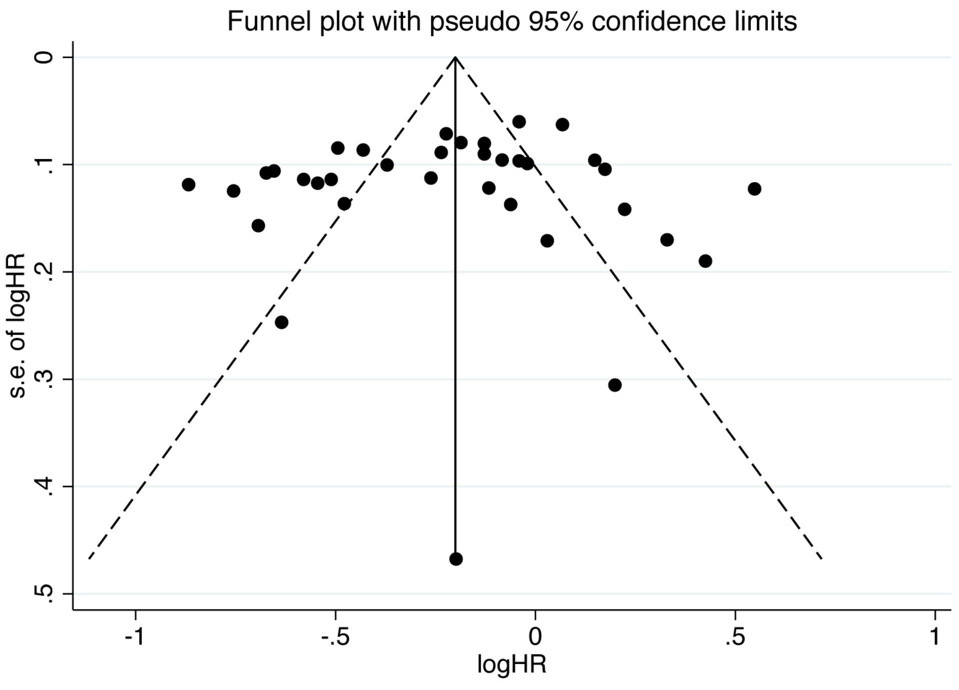

Supplementary Figure 9- Egger's test: Egger's test of OS (A) and PFS (B) from in included RCTs for calculative detection of association between the study effects and the study size.

A

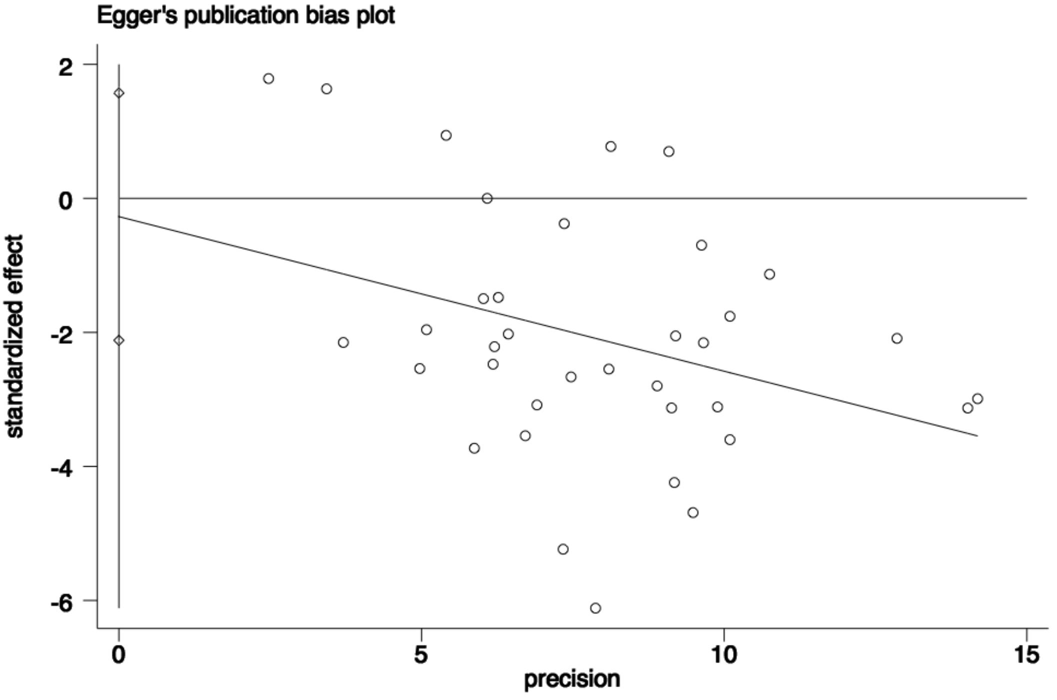

B

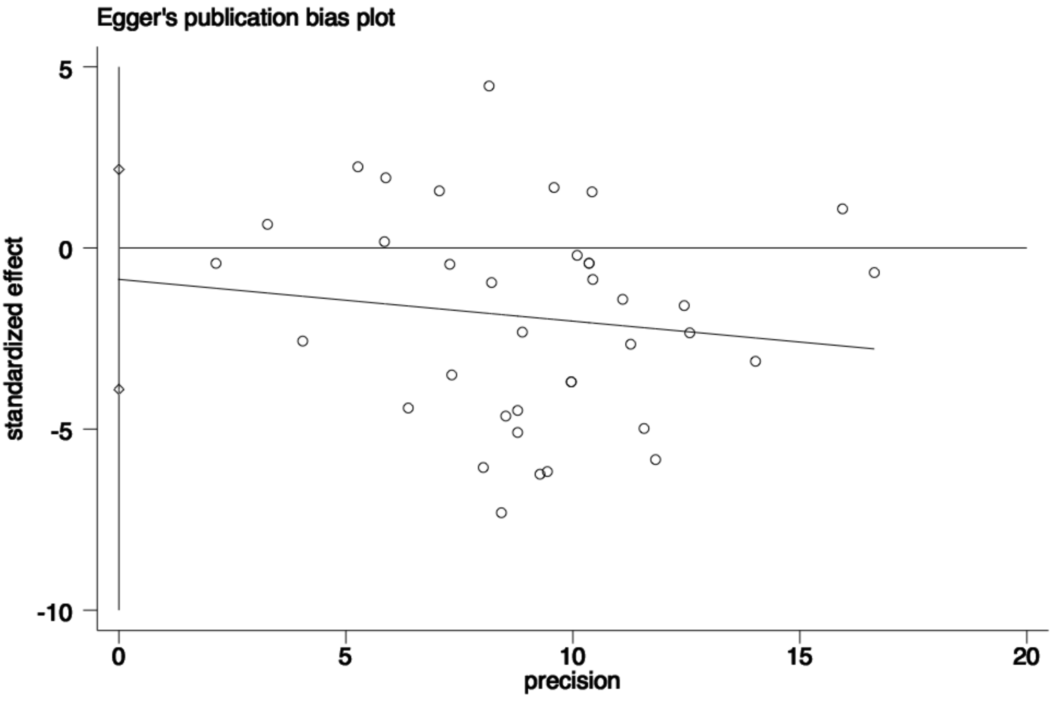

Supplementary Figure 10- Begg's test: Begg's test of OS (A) and PFS (B) from in included RCTs for calculative detection of association between the study effects and the study size.

A

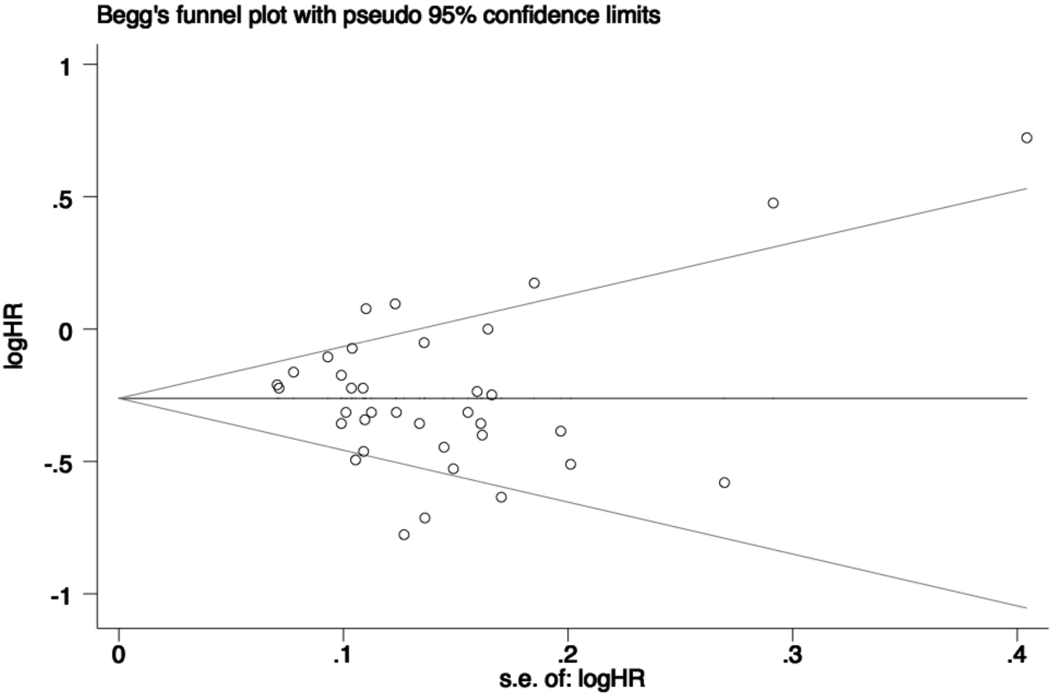

B

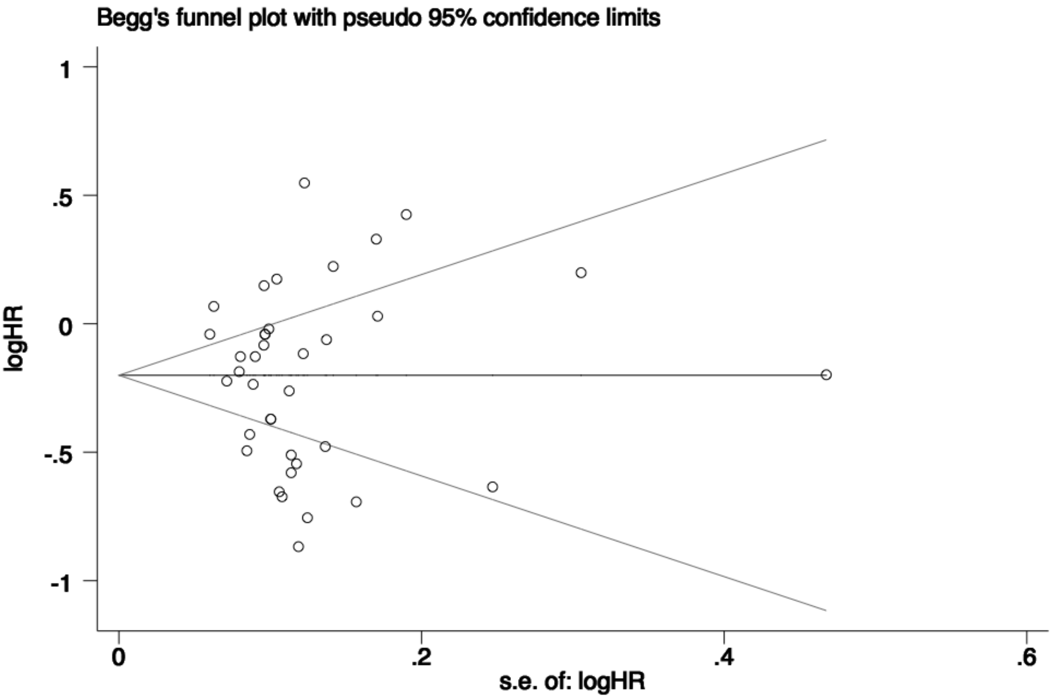

Supplement: Supplementary file 1 — Supplementary Material. [file 41598_2020_58674_MOESM1_ESM.pdf]
